# Evaluation of Left Ventricle Function by Regional Fractional Area Change (RFAC) in a Mouse Model of Myocardial Infarction Secondary to Valsartan Treatment

**DOI:** 10.1371/journal.pone.0135778

**Published:** 2015-08-20

**Authors:** Laura Castiglioni, Francesca Colazzo, Lucia Fontana, Gualtiero I. Colombo, Luca Piacentini, Elisa Bono, Giuseppina Milano, Serena Paleari, Annamaria Palermo, Uliano Guerrini, Elena Tremoli, Luigi Sironi

**Affiliations:** 1 Department of Pharmacological and Biomolecular Sciences, University of Milan, Milan, Italy; 2 Centro Cardiologico Monzino IRCCS, Milan, Italy; University of Louisville, UNITED STATES

## Abstract

**Aim:**

Left ventricle (LV) regional fractional area change (RFAC) measured by cardiac magnetic resonance (CMR) allows the non-invasive localization and quantification of the degree of myocardial infarction (MI), and could be applied to assess the effectiveness of pharmacological or regenerative therapies. Here we investigate the ability of RFAC to identify regional dysfunction and discriminate the effect of pharmacological treatment with valsartan, a selective antagonist of angiotensin II type 1 receptor, in a model of MI.

**Methods and Results:**

C57BL/6N mice, undergoing coronary artery ligation, were divided into two groups: untreated (MI) or treated with valsartan (MI+Val). Sham-operated mice were used as a control. Cardiac dimensions and function were assessed at baseline, 24 hours, 1 and 4 weeks post surgery by CMR and echocardiography. At sacrifice histology and whole-genome gene expression profiling were performed. RFAC was able to detect significant differences between treatment groups whereas the global ejection fraction was not. RFAC showed greater loss of regional contraction in remote non-infarcted myocardium in MI group than in MI+Val group. Consistently, in the same region MI+Val mice showed reduced myocyte hypertrophy, fibroblast proliferation, and fibrosis and modulation of target genes; in addition, left atrium volumes, appendage length and duct contraction were preserved.

**Conclusion:**

In this study, RFAC effectively estimated the degree of systolic dysfunction and discriminated the regions preserved by pharmacological treatment. RFAC index is a promising tool to monitor changes in LV contraction and to assess the effectiveness of therapeutic regimens in clinical settings.

## Introduction

Myocardial infarction (MI) leads to complex structural alterations (remodeling) involving both the infarcted and non-infarcted left ventricular (LV) myocardium characterized by chambers dilatation, myocyte loss, fibroblast proliferation, scar formation [1, 2], and compensatory hypertrophy. These conditions affecting LV wall stiffness are associated with worsening ventricular systolic function and abnormal cardiac remodeling, and are responsible for the increase in LV end-diastolic volume and pressure leading, consequently, to left atrial enlargement [3]. Clinical studies indicated fibrosis as the major independent predictive factor of adverse cardiac outcome [4, 5]. Nevertheless, there are no specific strategies based on tissue characterization of the myocardial wall in the therapeutic guidelines for post-ischemic heart failure. This lack of specific treatment stems from the absence of accurate clinical tools for phenotyping patients with heart disease and might lead to inappropriate therapies and possibly increased morbidity [6].

Imaging techniques play a central role in the diagnosis of acute myocardial infarction because of their ability to detect wall motion abnormalities or loss of viable myocardium. A number of reports proved the advantages of cardiac magnetic resonance (CMR) as reference tool for the non-invasive assessment of heart anatomy and function, advocating this approach as both an efficient method to monitor and an instrument to improve the therapeutic options [7–10]. CMR shows a high degree of accuracy in monitoring regional LV remodeling [11, 12], enabling objective quantification of subtle impairment in regional myocardial function [13, 14] and of pharmacological modulation on infarcted and viable myocardium.

Precise myocardial tissue characterization and a direct monitoring of treatment effects might be of great help in developing optimal therapeutic strategies for post-MI LV remodeling. Indeed, beneficial effects of pharmacological therapies on LV remodeling are associated with a better prognosis in MI patients: this makes maladaptive remodeling, occurring on remote non-infarcted myocardium, a therapeutic target for improving clinical outcome.

In the present study, we evaluated the power of regional fractional area change (RFAC) computed from CMR images in monitoring the effect of valsartan, an angiotensin II type 1 receptor (AT1R) antagonist, on LV remodeling in a mouse model of MI. RFAC is an index of LV regional wall motion and contraction [15] and is used to (*a*) assess qualitatively and quantitatively LV regional loss of function in infarcted and non-infarcted myocardium and (*b*) estimate drug effects occurring on myocardial wall motion. Imaging data were substantiated by tissue composition analysis and whole-genome gene expression profiling. Echocardiographic evaluation of left atrium (LA) volume was additionally used as index of LV performance and to indirectly monitor the LV diastolic function [16].

## Methods

### Animals

This study was carried out in strict accordance with the recommendations in the Guide for the Care and Use of Laboratory Animals of the National Institutes of Health. The protocol was approved by the Committee on the Ethics of Animal Experiments of the University of Milan (approval number 1242003—A 1371072003). Animals were fed *ad* libitum with standard chow and water. All surgery was performed under anesthesia, and all efforts were made to minimize the number of animals used and their suffering.

A total of 60 C57BL/6N female mice of 8–10 week old, weighting 18–20 g, purchased from Charles River (Calco, Italy), were used.

### Experimental protocol

Mice were anesthetized by intraperitoneal injection of a mixture of ketamine (75 mg/Kg) and medetomidine hydrochloride (1 mg/kg), endotracheally intubated in a supine position with a steel tube and ventilated with positive airway pressure (tidal volume of 140 μl at 150 breaths/min). After baseline CMR and echocardiography, MI was induced by left anterior descending (LAD) coronary artery ligation (n = 48), as previously described [17]. As control of surgery procedure, sham operated mice underwent thoracotomy and pericardiotomy without coronary artery ligation (n = 12). After surgery atipamezolo (2.5 mg/kg) was administered to encourage animal awakening, and then the animals were extubated, placed on an heating pad regulated to 38.5°C, and their breathing function monitored every 30 minutes until they were completely awake (usually in 2 hours).

After LAD ligation, surviving mice with an ejection fraction (EF) at 24-hour CMR within the range 35–45% were selected and randomized to vehicle or valsartan. Mice dying during the study or with unreliable measurements were replaced to obtain two final, equally-sized experimental groups of infarcted untreated (MI, n = 12) and treated (MI+Val, n = 12) animals.

Valsartan was administered in drinking water at the dose that did not influence systolic blood pressure (1 mg/Kg/day) [18, 19]. Drug was dissolved in distilled water at 10 mg/ml according to the manufacturer’s instructions and then diluted daily at the selected dose with a small amount (1 ml) of drinking water. After consumption of this small amount, the animals were given free access drinking water.

At 24 hours, 1 and 4 weeks after surgery, CMR was performed to evaluate LV parameters while echocardiography for LV, LA, and left atrial appendage (LAA) analysis (no LA and LAA quantification was possible by CMR for lack of both spatial and temporal resolution). Blood pressure was measured at the follow-up. Water consumption and body weight (BW) were carefully monitored during the follow-up. At sacrifice histological (n = 6/group) and genomic (n = 6/group) analyses were performed.

### Cardiac magnetic resonance

Induction of anesthesia was accomplished by exposing mice to 2% isoflurane (Merial, Toulouse, France) in 100% oxygen in an induction chamber. Mice were then fixed on a holder, anesthetized with inhaled 1% isoflurane in 100% oxygen, and placed into the 3.8 cm coil. Temperature was monitored rectally. The images were acquired using a 4.7T vertical-bore MR magnet (Bruker, Germany) and a retrospective gated cine gradient echo sequence with the following parameters: echo time (TE) 1.9 ms; repetition time (TR) 10 ms; field of view 4 × 4 cm^2^; acquisition matrix 128 × 128 pixels; slice thickness 1.3 mm; 6–8 axial slices spaced 1 mm to fully cover the LV.

The magnetic resonance images were analyzed using custom software implemented in Python along the lines of Franzosi *et al*. [12], to obtain LV global parameters as end-diastolic (LV EDV), end-systolic (LV ESV) and stroke volumes (LV SV), ejection fraction (LV EF) and posterior diastolic wall thickness (LV PWth). For the quantitative analysis of regional function and wall contraction, the LV cavity was divided into six 60° sectors (anterior, antero-septal, septal, lateral, posterior and inferior): RFAC was then computed for each segment as [(end diastolic area—end systolic area)/end diastolic area × 100] and used as index of regional endocardial wall motion [20]. Regional LV wall motion was displayed in a "bull’s eye" format, where the red tones represent lower and the green tones higher values. In this plot the inner circle represents the LV apex and the outer ones represent consecutive slices from apex to the base. To average the RFAC in mice with a different number of slices covering the LV, RFAC data were resampled using cubic spline interpolation to 10 slices for each of the six sectors. Regional LV thickness was obtained as previously reported [12].

### Echocardiography

Induction of anesthesia was accomplished by exposing the mice to 2% isoflurane in 100% oxygen in an induction chamber. Transthoracic echocardiography was then performed using 1% isoflurane in 100% oxygen to maintain heart rate (HR) ≥450 beats per minute (bpm). During acquisition animals were placed in the supine position on a heated (37°C) platform with integrated electrode pads used to obtain electrographic signals and HR. The mouse chest area was shaved and a warmed ultrasound gel applied to the thorax surface to optimize the visibility of the cardiac chambers. A Vevo 2100 echocardiography system (VisualSonics, Toronto, Canada) equipped with a MS400 30-MHz linear array transducer was used in these experiments.

LV cavity dimensions, ventricular function and mass were calculated on the M-mode parasternal short axis view. LA areas and longitudinal (supero-inferior, from the midpoint of the mitral annulus to the superior wall) and transversal diameters (medio-lateral, from the interatrial septum to the LA lateral wall, using the upper border of the LA-LAA duct as a marker) were measured on apical 4-chamber view, and minimum and maximum atrial volumes (LA Vmin, LA Vmax) were computed by Simpson’s rule. LA emptying fraction (LA EF) was calculated as the difference between LA Vmax and LA Vmin, divided by LA Vmax. On the same view the LAA maximum long axis (LAA length), as the mid-line curve between the LAA apex and duct during LV end-systole, was measured and the minimum and the maximum duct diameters assessed, in order to calculate duct diameter fractional shortening (LAA duct FS) as [(maximum—minimum) / maximum × 100] [21].

### Tail-cuff blood pressure and pulse measurements

Systolic arterial blood pressure and pulse were measured by tail-cuff plethysmography (BP-2000 Blood Pressure Analysis System; Visitech Systems, Apex, NC). Three days of training sessions were necessary to accustom animals to the procedure. Recording sessions (5 measurements each) were then made by a single investigator, at the baseline and at the different time points during the follow-up.

### Tissue collection and section preparation

To prepare specimens for histological analysis, abdominal aorta was cannulated and heart was arrested in diastole, with 2 mL of a solution of 0.1M CdCl_2_ and 1M KCl, and retrogradely perfused with 0.01 M phosphate saline buffer (PBS) and then with 4% (vol/vol) phosphate-buffered formalin for 10 min each time. Hearts were collected and LAA removed; tissues were postfixed in 4% phosphate-buffered formalin for 24 hours and separately embedded in paraffin. Consecutive 8 μm heart axial (from base to apex) and LAA sections were prepared.

For transcriptome analysis, hearts were perfused with PBS and the portion of the non-infarcted free wall corresponding to the posterior and inferior sectors was collected in RNAlater (Life Technologies, Carlsbad, CA) and stored at -80°C.

### Histological determination of infarct size

For collagen staining, deparaffinized and rehydrated heart sections were incubated in 0.1% Sirius Red Solution (Direct Red 80, Sigma-Aldrich, St. Louis, MO) in picric acid for 30 min, then washed, dehydrated (1 min each in 70%, 96%, and absolute ethylic alcohol, and then 10 min in xylene), and mounted with DPX mountant for microscopy. Images were acquired with a high-resolution digital camera using 1:1 macro-lens. Myocardial infarct size was determined on LV axial section (one for each mm on LV) along the apex-basis axis and expressed as percentage of the length of the infarct scar on the LV total circumferential length (using the average of endocardial and epicardial tracings) using the ImageJ v. 1.44o software.

### Immunostaining for morphometry, collagen, and cell proliferation

Immunofluorescence staining was performed on 3 tissue sections from each heart in order to assess myocyte cross-sectional area (MCSA), interstitial collagen fraction (ICF), and cell proliferation. Deparaffinized and rehydrated heart sections were incubated at room temperature in 10% normal goat serum (Dako, Glostrup, Denmark) in 0.01 M PBS and 0.1% Triton X-100 for 45 min. Primary and secondary antibodies were prepared in PBS and 0.1% Triton X-100. To detect cardiomyocytes, fibroblasts, myofibroblasts, collagen I deposition, and cell proliferation the sections were incubated overnight at 4°C respectively with anti-α-sarcomeric actin (1:800), anti-vimentin (1:1500), anti-α-SMA mouse monoclonal antibodies (1:400; all previous from Sigma-Aldrich, St. Louis, MO), anti-collagen type I (1:50, Rockland, Gilbertsville, PA), and anti- Ki-67 rabbit polyclonal antibodies (1:100, Novocastra Laboratories, Newcastle, UK). Appropriate secondary antibodies (Alexa 555 goat anti-mouse IgM or IgG [1:600] and Alexa 488 goat anti-rabbit [1:400], all from Invitrogen, Carlsbad, CA) were applied for 2 h at room temperature. For nuclear staining, the sections were incubated with Hoechst 33258 (2.5 g/ml; Invitrogen) in PBS for 15 min. Images were acquired at fixed exposure times using an inverted fluorescence microscope (Axiovert 200; Zeiss, Jena, Germany) equipped with the Axiovision v. 3.1 software (Zeiss).

MCSA was assessed on LV tissue sections, double-labeled with anti-α-sarcomeric actin and anti-collagen type I: cardiomyocytes cut along the short axis, showing a circular profile and a visible nucleus were selected and their area was traced. ICF was measured within the LV non-infarcted zone and LAA and expressed as percentage of area occupied by collagen on total tissue area. Proliferating fibroblasts in LV remote non-infarcted myocardium were detected by double labeling with anti-Ki-67 as marker of proliferation and anti-vimentin as marker of fibroblasts. Cell proliferation was expressed as percentage of Ki-67 positive cells over the total number of cells (counting the nuclei); the rate of fibroblasts proliferation was expressed as a percentage of vimentin/Ki-67 double-positive cells over vimentin positive cells. Measurements were performed on 5 and 10 fields per section (220μm × 165 μm) for ICF and cell proliferation, respectively, whereas MCSA was based on 100 measured cardiomyocytes. All quantitative analyses were performed using Photoshop CS6 by an investigator blinded for the experimental groups.

### Gene Expression Analysis

Total RNA was isolated from LV non-infarcted free wall using the TRIzol Reagent (Life Technologies) and further purified using silica-membrane RNeasy spin columns (Qiagen, Hilden, Germany), following the manufacturers' instructions. Genomic DNA contamination was removed by TURBO DNA-free DNase (Life Technologies) treatment. Messenger RNA was reverse transcribed, labeled, and linearly amplified using the Total Prep RNA Amplification Kit (Life Technologies), and then hybridized to MouseWG-6 v.2 Expression BeadChip microarrays (Illumina, San Diego, CA), according to the manufacturers' instructions. Signal detection was performed using a high resolution confocal scanner iScan, and quantitation and quality control were performed with the GenomeStudio v. 1.9.0 software (Illumina).

Data adjustment, probe filtering, gene annotation and primary statistical analysis were performed using BRB-Array Tools v. 4.3.2, developed by Dr. Richard Simon and BRB-Array Tools Development Team, and software packages of Bioconductor v. 2.12 and R v. 3.0.1. Data variance stabilizing transformation (VST) and robust spline normalization (RSN) were conducted with the *lumi* R package. To focus on the most informative probes and genes, we used the following filtering criteria: a probe was excluded if (*a*) the 33^th^ percentile of intensities was less than 125 (corresponding to a detection p-value less than 0.01, as reported by GenomeStudio) and/or (*b*) the p-value of the log-variation was greater than 0.05; (*c*) multiple probes were reduced to one per gene symbol by using the most variable probe measured by interquartile range across arrays. The number of genes that passed these filtering criteria was 8633. Differentially expressed genes among the three classes were identified by using a multivariate permutation test, based on 1000 random permutations, to provide 80% confidence (CI) that the false discovery rate (FDR) was less than 10%. The test statistics used was F-statistics for each gene; nonetheless, the multivariate permutation test is non-parametric and does not require the assumption of Gaussian distributions. We then performed a pairwise comparison (*t*-test) between pairs of classes, setting α = 0.01 for significantly different gene expression. Finally, to focus on the most meaningful differences, we filtered out those genes with a mean fold change (FC) ≤ ±1.2 in any of the two relevant post-hoc comparisons (*i*.*e*., MI *vs*. Sham and MI *vs*. MI +Val).

To identify clusters of genes with distinct expression patterns between the three conditions, we used the CLICK (CLuster Identification via Connectivity Kernels) clustering algorithm, implemented in the EXPANDER v. 6.3 (EXPression ANalyzer and DisplayER) software package [22] with the default homogeneity parameter ≥ 0.65 for a balanced intra-cluster similarity and inter-cluster separation. Functional enrichment analysis was performed using the TANGO (Tool for ANalysis of GO enrichments) algorithm, integrated in EXPANDER. The algorithm performs hyper-geometric enrichment tests for Gene Ontology (GO) terms (in *biological process* and *molecular functions* ontologies) and corrects for multiple testing by bootstrapping (1000 bootstraps), estimating the empirical p-value distribution for the evaluated sets. A functional class was considered significantly enriched in a cluster if its corrected p-value was lower than 0.05.

To identify genes whose expression was significantly related to RFAC, we computed a statistical significance level for each gene using the Pearson’s correlation test. These p-values were then used in a multivariate permutation test in which the RFACs were randomly permuted among arrays. We used the multivariate permutation test to provide 80% confidence that the FDR was less than 10%.

The data discussed in this publication have been deposited in NCBI's Gene Expression Omnibus [23] and are accessible through GEO Series accession number **GSE68426**.

### Statistical analysis

Statistical analyses were performed using R: A Language and Environment for Statistical Computing (R Core Team, R Foundation for Statistical Computing, Vienna, Austria, 2013, http://www.R-project.org). Values are shown as mean ± SEM (standard error of the mean), unless indicated; p<0.05 was considered significant. For measurements at the 24-hour time point, *i*.*e*. before pharmacologic treatment, we performed an ANOVA with Helmert contrast comparing (*a*) the sham group to the average of the MI and MI+Val groups, to assess that surgery was effective, and (*b*) MI group to MI+Val group, to assess that the assignment was unbiased. For measurements after the pharmacologic treatment (1 and 4 weeks), ANOVA with "treatment" contrast was performed to compare MI group either with MI+Val or sham group. Repeated measures ANOVA with post-hoc Holm adjustment was performed on the sham group to assess time constancy. RFAC values of MI and MI+Val groups were compared, sector by sector and slice by slice (on the resampled 10-slice space), using unpaired *t*-test. Mid-apical RFAC values were also averaged by sector excluding the apex slice (unreliable due to apex movement, which causes partial volume artifacts) and the three at the base end of the heart not affected by infarction. Finally, Kaplan-Meier survival curves and the log-rank test were used to compare the mortality rate among groups after randomization to treatment with valsartan.

## Results

### Mortality, body weight and systolic blood pressure

**Fig 1:**
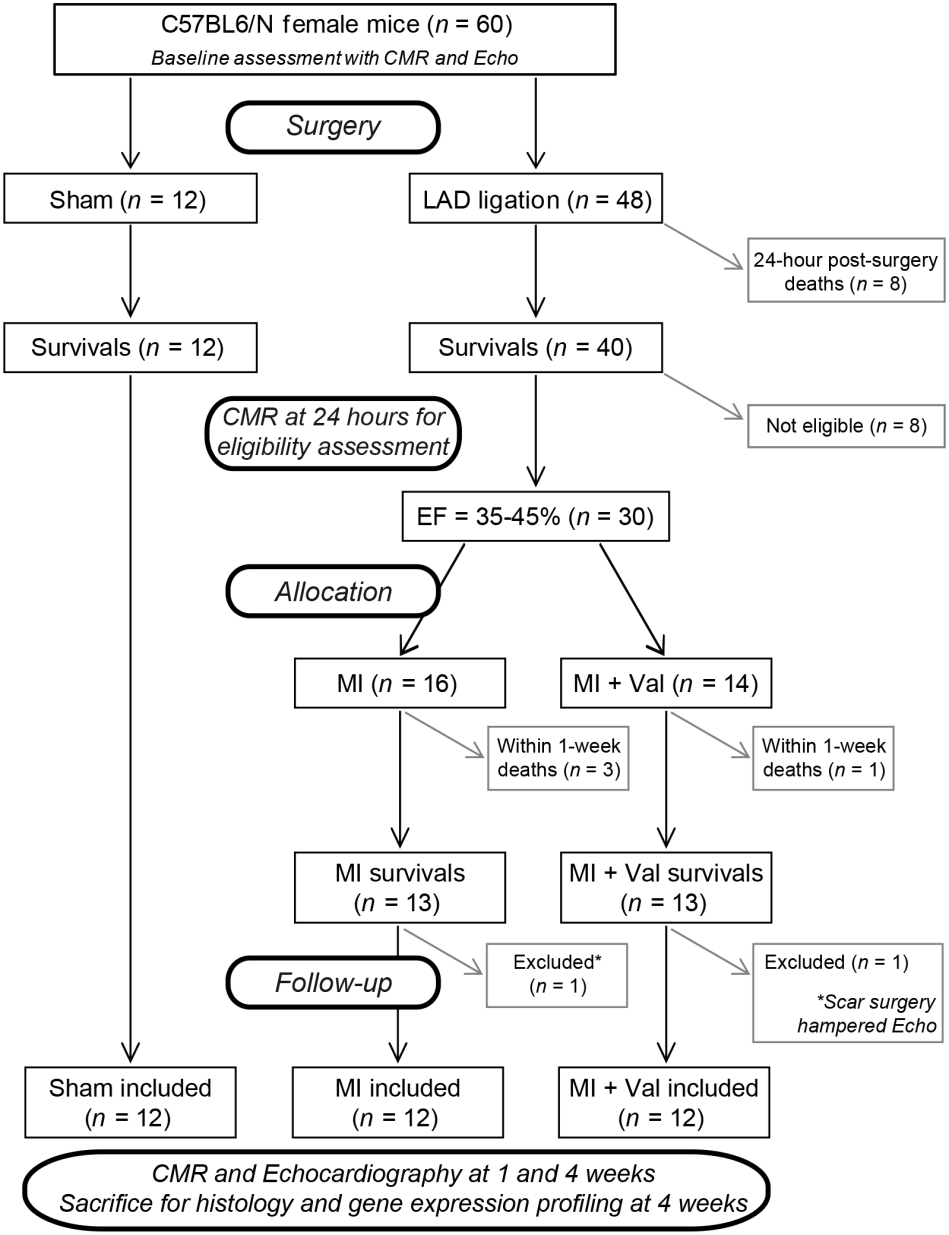
Flow chart showing the experimental protocol with the number of animals used, died and included in the study.

Of the 48 mice that underwent LAD ligation (Fig 1) 8 died before 24 hours, 10 were excluded because their EF was out of 35–45% range at 24-hour CMR, 2 were excluded because surgery scar prevented correct Echo measurements, and 4 died within 1 week after randomization (3 MI and 1 MI+Val). No other death was observed in the remaining follow-up period. All 12 sham-operated mice survived during the follow-up period. No significant differences in mortality rate were observed among the three study groups after randomization to treatment.

Mice BW, following an initial decrease immediately after surgery, tended to increase during the follow-up period without significant differences among the experimental groups at 4 weeks (sham: 21.6 ± 0.3 g; MI: 22.8 ± 0.4 g; MI+Val: 22.3 ± 0.4 g).

No significant difference was found in heart rate (ranging 590–640 bpm) and in systolic blood pressure among the three groups at follow-up (baseline: 102 ± 4 mmHg; 4 weeks post-surgery: sham: 110 ± 2 mmHg; MI: 111 ± 7 mmHg; MI+Val: 118 ± 6 mmHg).

### Cardiac function

During the follow-up period ventricular parameters in sham-operated mice remained unchanged compared to baseline, whereas in MI and in MI+Val mice LV EDV and LV ESV increased steadily exhibiting severe LV dilatation and a large significant reduction in LV EF (Table 1). Nevertheless, LV SV remained almost unchanged at follow-up compared to baseline (baseline: 27 ± 1 μl; 4 weeks post-surgery sham: 33 ± 1 μl; MI: 30 ±2 μl; MI+Val: 27 ±1 μl).

**Table 1:** Left ventricular global and regional function.

	Mice	iRFAC	pRFAC	ipRFAC	LV EF	LV EDV	LV ESV
		(%)	(%)	(%)	(%)	(μl)	(μl)
**Baseline**	12	69 ± 1	75 ± 1	72 ± 1	65 ± 1	42 ± 2	15 ± 1
**24h**							
Sham	12	71 ± 1	77 ± 1	74 ± 1	65 ± 1	38 ± 1	13 ± 1
MI and MI+Val	24	32 ± 2^§§§^	31 ± 3^§§§^	31 ± 2^§§§^	37 ± 1^§§§^	56 ± 1^§§§^	35 ± 1^§§§^
MI	12	29 ± 3	27 ± 3	28 ± 3	37 ± 1	58 ± 2	37 ± 1
MI+Val	12	36 ± 4	35 ± 4	35 ± 4	38 ± 1	53 ± 2	33 ± 1
**1w**							
Sham	12	74 ± 2	77 ± 1	75 ± 2	69 ± 1	41 ± 1	13 ± 1
MI	12	21 ± 3^§§§^	18 ± 2^§§§^	20 ± 3^§§§^	29 ± 2^§§§^	96 ± 6^§§§^	69 ± 6^§§§^
MI+Val	12	34 ± 4**	31± 5*	32 ± 5**	34 ± 2	80 ± 4*	53 ± 4*
**4w**							
Sham	12	74 ± 3	80 ± 2	77 ± 2	68 ± 2	47 ± 1	15 ± 1
MI	12	22 ± 3^§§§^	17 ± 2^§§§^	20 ± 2^§§§^	26 ± 2^§§§^	117 ± 8^§§§^	87 ± 7^§§§^
MI+Val	12	38 ± 4**	30 ± 5**	34 ± 4**	32 ± 2	90 ± 5**	63 ± 5**

Cardiac magnetic resonance evaluation of regional and global left ventricle function at the baseline and in sham, MI and MI+Val mice included in the study during the follow-up period (24 hours, 1 and 4 weeks). Values are presented as mean ± SEM.

* p<0.05 and

** p<0.01 *vs*. MI.

^§§§^ p<0.001 *vs*. Sham.

As shown in Table 1, when compared to MI group, MI+Val mice showed an attenuated LV enlargement as indicated by significantly lower LV EDV and ESV. In addition, LV PWth, which was significantly increased at the follow-up by myocardial infarction (1.06 ± 0.04 mm and 0.91 ± 0.03 mm in MI and sham, respectively, *p*<0.05), was attenuated by treatment (0.82 ± 0.04 mm, *p*<0.01 MI *vs*. MI+Val).

Echocardiography analyses of LV structure and function (S1 Table) confirmed CMR data, showing additionally an increase in LV mass at 4 weeks post-surgery in MI-mice (175 ± 14 mg) compared to sham (94 ± 14 mg, *p*<0.001 MI *vs*. sham) that was attenuated by treatment with valsartan (135 ± 9 mg, *p*<0.01 MI *vs*. MI+Val).

### Regional LV function—Bull’s eye representation

**Fig 2:**
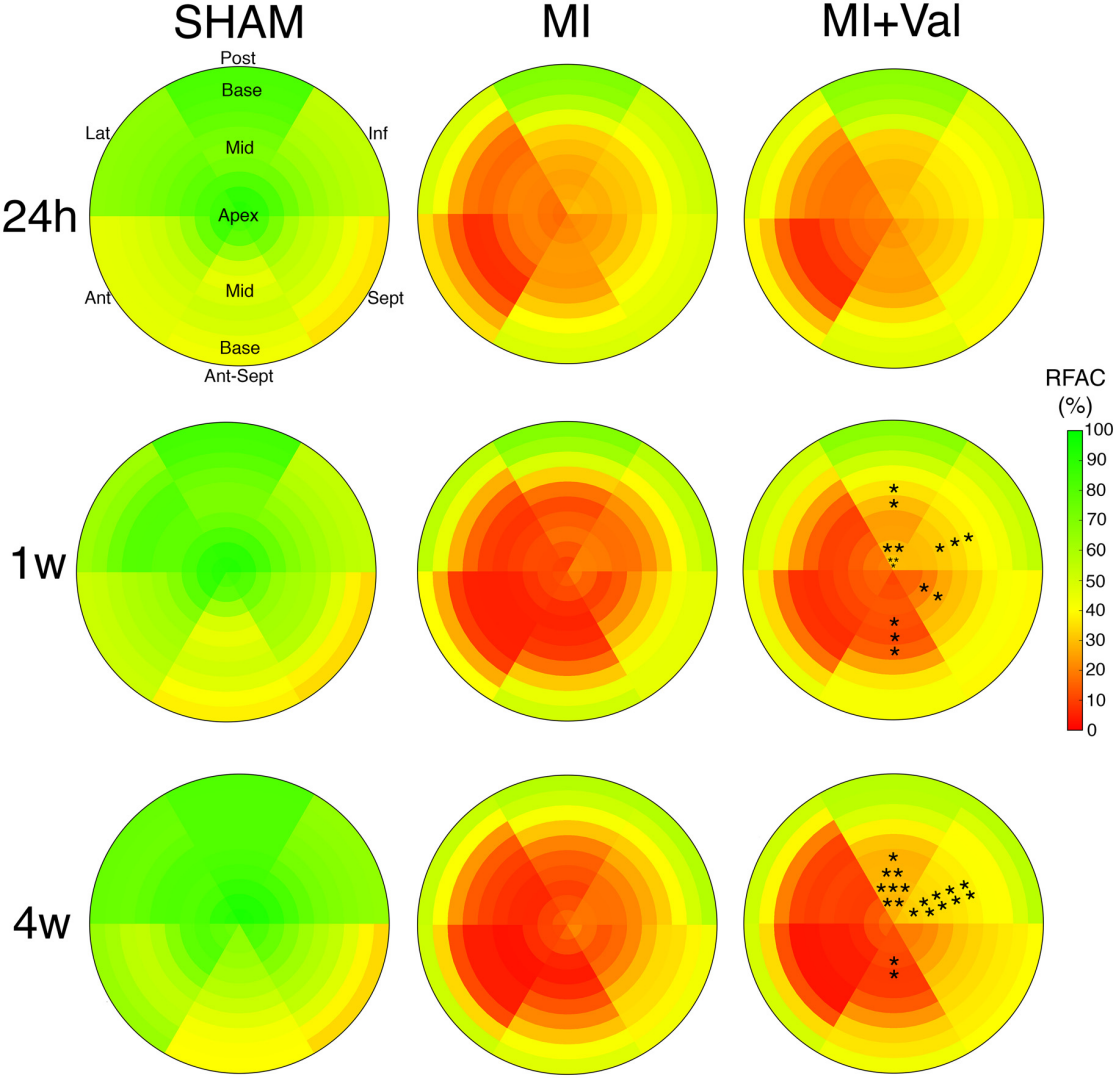
Bull’s eye representations of mean regional fractional area change (RFAC) in sham, MI and MI+Val groups at 24 hours, 1 and 4 weeks after MI (n = 12/group). Slices from LV apex to the base are shown from the inner to the outer circle, whereas red and green tones indicate lower and higher RFAC values (the color bar on the right shows the corresponding scale). • for 0.05<p<0.1, * for p<0.05, ** for p<0.01. Lat: lateral; Post: posterior; Inf: inferior; Sept: septal; Ant-Sept: Antero-septal; Ant: anterior.

During the follow-up, anterior myocardial infarction resulted in acute loss of LV systolic function and progression of LV impairment. At 24 hours after surgery, bull’s eye representation of regional LV contractile function showed a reduced contraction of anterior, antero-septal and lateral sectors from apex to mid papillary region (mid-apical in the following) in infarcted mice (MI and MI+Val) compared to sham group (Fig 2). At 1-week post surgery, when the remodeling process was ongoing, the progression of LV impairment was evident in both infarcted groups. RFAC discriminated regional differences in MI+Val compared to MI mice, showing at the mid-apical level a significant protective effect in terms of contraction property exerted by valsartan on the posterior, inferior, septal, and antero-septal sectors (Fig 2). The higher degree of preserved mid-apical function observed at 1 week in the treated group became more significant at 4 weeks, with a predominant localization to the posterior and inferior mid-apical sectors (Fig 2).

Results of the non-infarcted free wall are shown in Table 1, where averages of mid-apical RFAC of the inferior (iRFAC) and posterior (pRFAC) sectors and their mean (ipRFAC) are presented. These indices showed significant difference between MI+Val and MI group in regional function of remote non-infarcted tissue; on the contrary, global EF could not discriminate between the groups (Table 1). Power analysis showed that sample sizes of 12 per group achieved 85% power to detect a difference of 14.0 in ipRFAC between MI and MI+Val groups at 4 weeks (Table 1), with the observed group standard deviations of 10.4 and 17.3 and with a significance level (α) of 0.05 in a pairwise comparison.

### Left atrium remodeling

LA and LAA parameters in sham-operated mice remained almost unchanged from baseline to follow-up (Table 2). On the contrary, LA remodeling was evident 1 week after surgery in both infarcted groups with an increase of volumes in comparison with sham group and no significant differences between MI and MI+Val group. At 4 weeks LA Vmin was significantly greater in MI compared to MI+Val group.

**Table 2:** LA and LAA remodeling following Myocardial Infarction.

	Mice	LA Vmin	LA Vmax	LA EF	LAA length	LAAduct FS
		(μl)	(μl)	(%)	(mm)	(%)
**Baseline**	12	2.0 ± 0.1	4.7 ± 0.4	57.2 ± 1.7	3.5 ± 0.1	47.9 ± 2.3
**24h**						
sham	12	2.1 ± 0.1	4.6 ± 0.3	53.6 ± 1.7	3.4 ± 0.1	51.4 ± 1.6
MI and MI+Val	24	2.9 ± 0.3	5.5 ± 0.4	48.9 ± 2.4	3.6 ± 0.1	41.3 ± 1.3^§§§^
MI	12	2.8 ± 0.6	5.6 ± 1.0	49.9 ± 2.4	3.5 ± 0.2	43.6 ± 1.8
MI+Val	12	2.9 ± 0.4	5.5 ± 0.4	48.3 ± 4.3	3.8 ± 0.1	38.9 ± 1.6
**1w**						
sham	12	2.3 ± 0.1	4.9 ± 0.3	52.7 ± 1.6	3.5 ± 0.1	48.2 ± 3.1
MI	12	4.2 ± 0.3^§§§^	7.6 ± 0.4^§§§^	45.6 ± 2.9^§^	4.2 ± 0.2^§§§^	32.2 ± 6.2^§§^
MI+Val	12	3.7 ± 0.2	7.5 ± 0.6	50.8 ± 1.6	4.1 ± 0.1	30.8 ± 3.7
**4w**						
sham	12	2.6 ± 0.2	5.6 ± 0.4	54.3 ± 1.2	3.5 ± 0.1	43.3 ± 2.0
MI	12	5.2 ± 0.7^§§§^	8.8 ± 1.0^§§§^	43.1 ± 2.1^§§§^	4.8 ± 0.2^§§§^	19.2 ± 3.8^§§§^
MI+Val	12	3.6 ± 0.3*	7.3 ± 0.4	50.1 ± 1.7**	4.0 ± 0.1***	40.6 ± 2.0***

Echocardiographic evaluation of LA and LAA at baseline and during the follow-up (24 hours, 1 and 4 weeks) in sham, MI and MI+Val mice included in the study.

Left Atrial Minimum Volume (LA Vmin); Left Atrium Maximum Volume (LA Vmax); Left Atrial Emptying Fraction (LA EF); Left Atrial Appendage Maximum long axis (LAA length); Left Atrial Appendage duct diameter fractional shortening (LAA duct FS). Values are presented as mean ± SEM.

* p<0.05,

** p<0.01, and

*** p<0.001 *vs*. MI.

^§^ p<0.05,

^§§^ p<0.01 and

^§§§^ p<0.001 *vs*. Sham.

**Fig 3:**
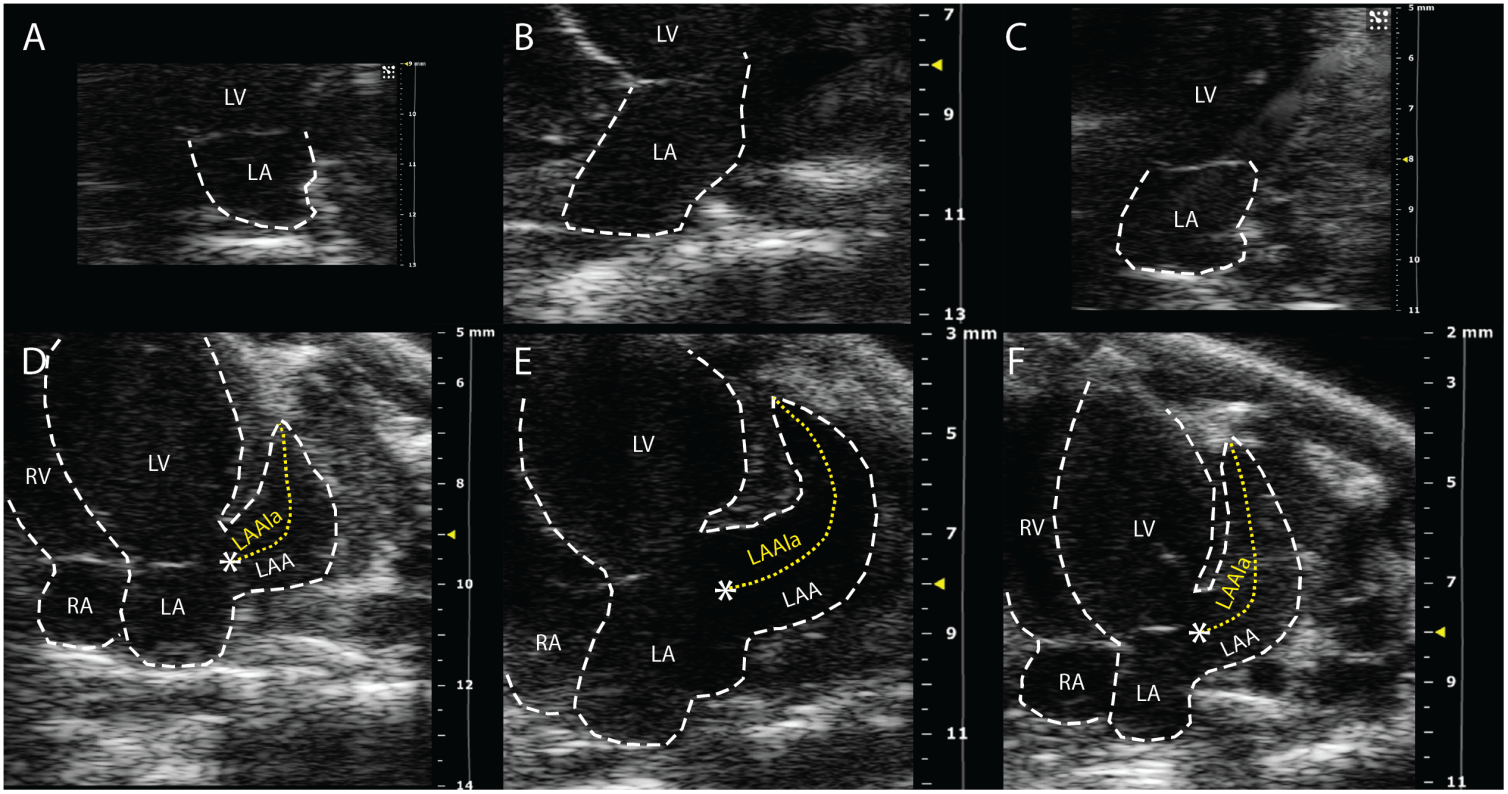
Echocardiographic evaluation of left atrium (LA) and left atrium appendage (LAA) by apical 4-chamber views, 4-weeks after surgery, representative images. In upper panels, LA maximum area is shown in sham (A), MI (B) and MI+Val (C) mice. In lower panels, LAA long axis is shown in sham (D), MI (E) and MI+Val (F) mice. LAAla: LAA long axis; LV: left ventricle; RA: right atrium.

Concurrently, LAA length increased at week 1 and 4 in MI compared to sham group and, LAA duct contraction was significantly and progressively lost. At the follow-up, treatment significantly preserved both parameters (Table 2, Fig 3).

### Histopathology

**Fig 4:**
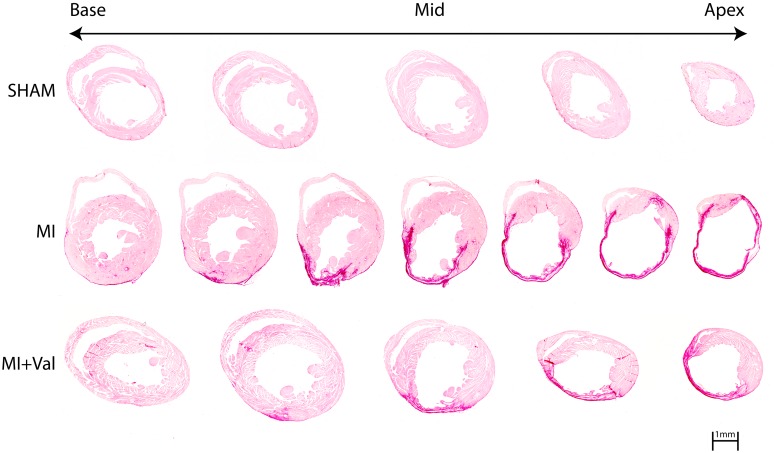
Representative photomicrographs of infarct size evaluated from the base (left) to the apex (right) by Sirius red staining measured at 4 weeks after surgery in sham, MI and MI+Val groups. Scale bar: 1 mm.

Coronary artery ligation resulted in infarction with loss of cardiomyocytes and scar formation. Four weeks after surgery, infarct size, determined by Sirius red staining, was clearly greater in MI than in MI+Val mice (39.3 ± 2.2% *vs*. 32.9 ± 1.9%, *p*<0.05). No damage was observed in sham mice (Fig 4).

**Fig 5:**
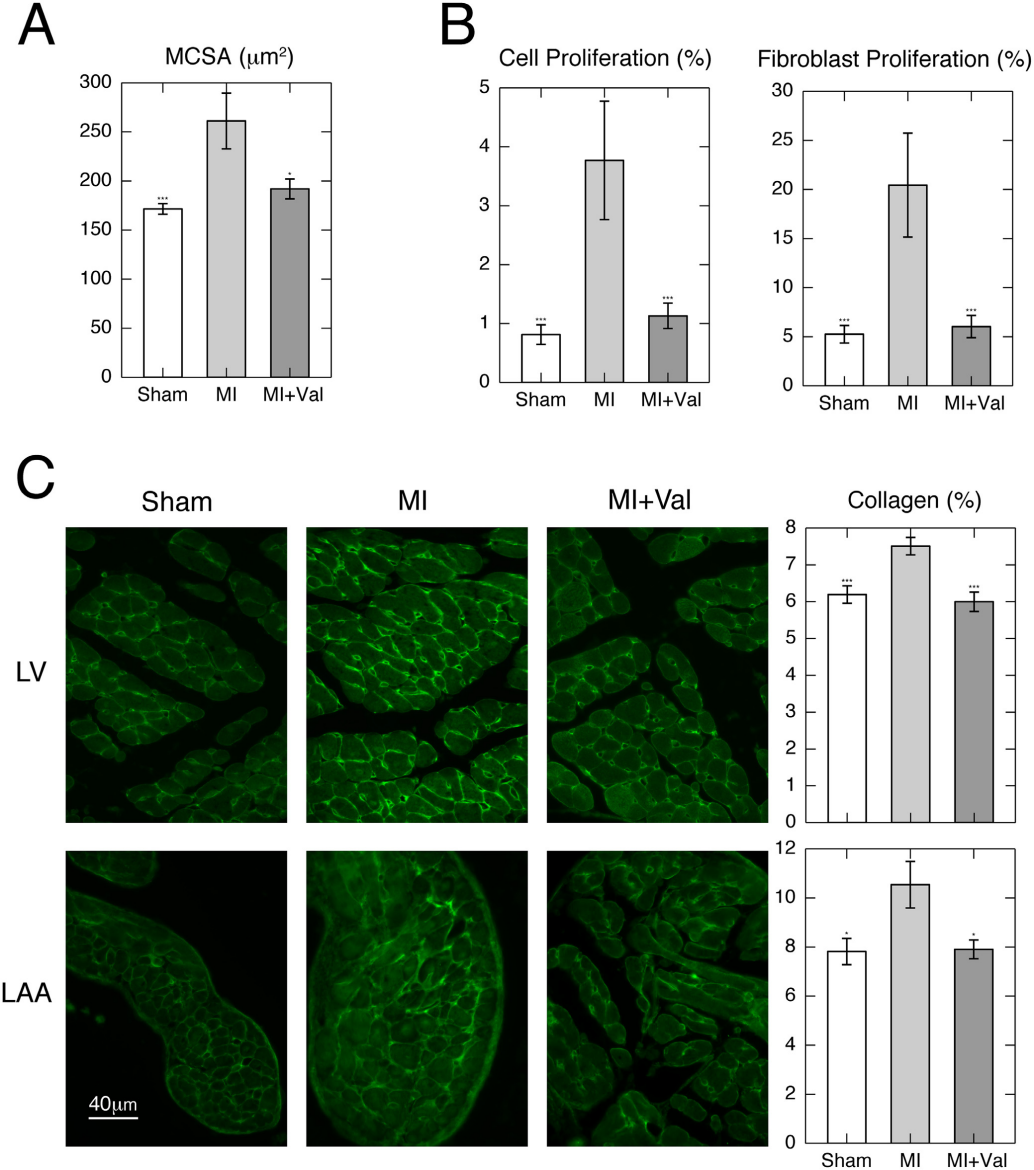
(**A**) Myocyte cross-sectional area (MCSA) in the remote left ventricle (LV) in sham and infarcted groups (n = 6/group). (**B**) Cell proliferation measured as KI-67 positivity and proliferating fibroblasts detected by double labeling with KI-67 and vimentin. Cells proliferation was expressed as percentage of KI-67 positive cells over total cell number (nuclei), whereas fibroblasts proliferation was expressed as percentage of vimentin/KI-67 double-positive cells over vimentin positive cells. (**C**) Representative immunofluorescence and quantification of collagen type I staining of the non-infarcted LV (upper panels) and of appendage (lower panels) in sham, MI and MI+Val mice at 4 weeks post-surgery. Interstitial collagen fraction was measured as the percentage of area occupied by collagen on total tissue area. Values are mean ± SEM. * *p*<0.05, *** *p*<0.001 vs. MI. (n = 6/group).

MCSA increased 1.5-fold in MI *vs*. sham hearts, whereas valsartan treatment preserved it almost entirely (MI *vs*. sham *p*<0.001; MI *vs*. MI+Val *p*<0.05; Fig 5A). Immunofluorescence analysis for Ki-67 showed an increased cell and fibroblast proliferation in non-infarcted myocardium in MI compared to sham group, which is almost completely abrogated by treatment with valsartan (MI *vs*. sham and MI *vs*. MI+Val *p*<0.001; Fig 5B).

ICF showed a decreased collagen content in treated mice versus MI (*p*<0.001, MI *vs*. sham and MI *vs*. MI+Val). LAA collagen content in MI group increased 1.4-fold compared to sham, while treatment maintained it at the sham level (MI *vs*. sham and MI *vs*. MI+Val *p*<0.05; Fig 5C).

### Gene expression

To find which genes were significantly modulated by valsartan, we performed a genome-wide gene expression analysis comparing the 3 groups at the 4-week time point. The number of significant genes by controlling the proportion of false discoveries, at a FDR<0.1 with a CI = 80% in a multivariate permutation F test (*p*<0.002), was 248. The number of genes with a significant difference (*p*<0.01, FC ≥ ±1.2) in any of the two relevant post-hoc pairwise comparisons (MI *vs*. sham and MI *vs*. MI+Val) was 174 (out of 248): of these, respectively 27 and 53 genes were significantly up- or down-regulated by valsartan with respect to MI mice (S2 Table).

**Fig 6:**
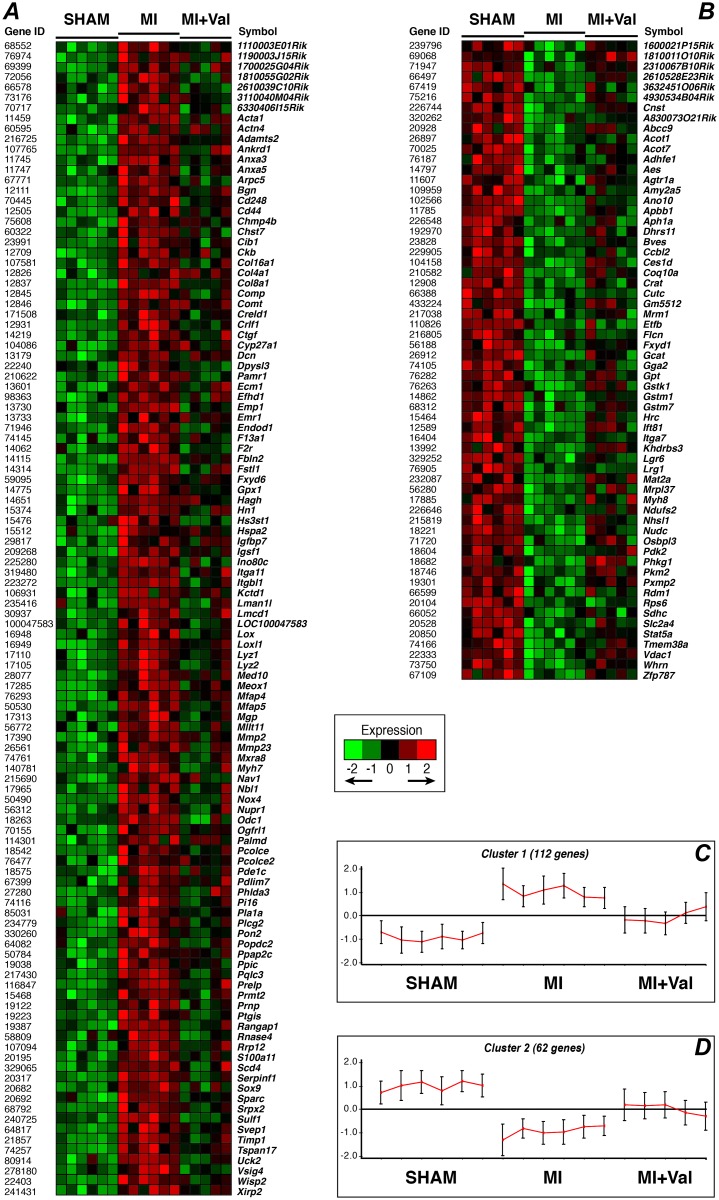
Clusters of genes significantly modulated by valsartan treatment after myocardial infarction. (***A***) Heatmap and (***C***) mean expression pattern of cluster 1, *i*.*e*. genes overexpressed in MI *vs*. sham group and down-regulated in MI+Val *vs*. MI mice. (***B***) Heatmap and (***D***) mean expression pattern of cluster 2, *i*.*e*. genes that were decreased in MI *vs*. sham group and up-regulated in MI+Val *vs*. MI mice. In the heatmaps, normalized expression values are mean-centered and represented with a green, black, and red color scale (green indicates below, black equal to, and red above the mean). In the graphs ***B*** and ***D***, each cluster is represented by the mean expression pattern over all the genes assigned to it for each sample; error bars denote ± 1 SD (n = 6/group).

The CLICK algorithm identified 2 clusters of genes, with an overall average homogeneity of 0.834 (Fig 6): (1) 112 genes that were significantly overexpressed in MI in comparison with sham group and down-regulated in MI+Val with respect to MI mice (Fig 6A and 6C), and, conversely, (2) 62 genes that were decreased in MI compared to sham group and up-regulated in MI+Val with respect to MI mice (Fig 6B and 6D). Gene set enrichment analysis of these two clusters (Table 3) indicated that valsartan significantly modulates a number of biological processes in MI mice. In particular, cluster 1 was significantly enriched with genes belonging to the GO categories of regulation of cell death and/or cell proliferation, cardiovascular development, integrin-mediated signaling pathway, extracellular matrix (ECM) organization and/or binding, response to stress, and calcium binding. Cluster 2 was enriched with genes belonging to amino acid metabolism and establishment of localization (*i*.*e*. the directed movement of a cell, a protein complex or organelle to a specific location), and genes with relevant transferase activity, for instance, for the glutathione metabolism.

**Table 3:** GO enrichment analysis of differentially expressed gene clusters.

GO	GO term	p_*corr*_	Gene List
***Cluster_1***			
BP	regulation of cell death	0.001	*Ptgis*, *Plcg2*, *Nox4*, *Anxa5*, *Prmt2*, *Ankrd1*, *Cd44*, *Ctgf*, *Phlda3*, *Gpx1*, *Nupr1*, *Actn4*, *Crlf1*, *Mllt11*, *F2r*, *Timp1*, *Prnp*, *Sox9*, *Mmp2*, *Comp*
	cellular component organization	0.002	*Prmt2*, *Lox*, *Nav1*, *Efhd1*, *Kctd1*, *Sulf1*, *Gpx1*, *F2r*, *Sox9*, *Mmp2*, *Acta1*, *Dpysl3*, *Nox4*, *Mgp*, *Anxa5*, *Hspa2*, *Xirp2*, *Cd44*, *Pdlim7*, *Mfap5*, *Ctgf*, *Col4a1*, *Nbl1*, *Nupr1*, *Actn4*, *2610039C10Rik*, *Arpc5*, *Prnp*, *Adamts2*, *Comp*
	system development	0.001	*Plcg2*, *Odc1*, *Lox*, *Nav1*, *Efhd1*, *Sulf1*, *Gpx1*, *Col8a1*, *F2r*, *Timp1*, *Sox9*, *Mmp2*, *Acta1*, *Ecm1*, *Dpysl3*, *Nox4*, *Mgp*, *Srpx2*, *Ankrd1*, *Cd44*, *Myh7*, *Ctgf*, *Col4a1*, *Sparc*, *Nbl1*, *Serpinf1*, *Nupr1*, *Igfbp7*, *Adamts2*, *Comp*
	regulation of angiogenesis	0.025	*Ptgis*, *Serpinf1*, *Srpx2*, *Anxa3*, *Sulf1*, *Ecm1*
	regulation of developmental process	0.002	*Ptgis*, *Mgp*, *Srpx2*, *Anxa3*, *Ankrd1*, *Sulf1*, *Cd44*, *Pdlim7*, *Ctgf*, *Nbl1*, *Gpx1*, *Serpinf1*, *Nupr1*, *Med10*, *Timp1*, *Palmd*, *Sox9*, *Ecm1*, *Dpysl3*
	cardiovascular system development	0.004	*Nox4*, *Gpx1*, *Srpx2*, *Col8a1*, *Lox*, *Ankrd1*, *Myh7*, *Col4a1*, *Ctgf*, *Sox9*, *Mmp2*, *Ecm1*
	skeletal muscle fiber development	0.035	*Gpx1*, *F2r*, *Col4a1*, *Acta1*
	muscle organ development	0.024	*Gpx1*, *Nupr1*, *Ankrd1*, *F2r*, *Myh7*, *Col4a1*, *Acta1*
	anatomical structure formation involved in morphogenesis	0.041	*Gpx1*, *Srpx2*, *Col8a1*, *Meox1*, *Cd44*, *Col4a1*, *Ctgf*, *Sox9*, *Mmp2*, *Acta1*, *Ecm1*
	tissue development	0.002	*Mgp*, *Ankrd1*, *Sulf1*, *Myh7*, *Cd44*, *Ctgf*, *Col4a1*, *Gpx1*, *Nupr1*, *F2r*, *Sox9*, *Mmp2*, *Acta1*, *Ecm1*, *Adamts2*, *Comp*
	cell adhesion	0.002	*Srpx2*, *Igfbp7*, *Col8a1*, *Itgbl1*, *Wisp2*, *Svep1*, *Cd44*, *Mfap4*, *Itga11*, *Col16a1*, *Ctgf*, *Sox9*, *Comp*
	integrin-mediated signaling pathway	0.012	*Cib1*, *Itgbl1*, *Itga11*, *Ctgf*, *Adamts2*
	regulation of cellular component movement	0.002	*Nox4*, *Nbl1*, *Srpx2*, *Actn4*, *Anxa3*, *Sulf1*, *Timp1*, *F2r*, *Sox9*, *Ecm1*, *Dpysl3*
	regulation of peptidase activity	0.002	*Gpx1*, *Serpinf1*, *Pi16*, *Timp1*, *F2r*, *Cd44*, *Pcolce*, *Ctgf*, *Ecm1*, *Pcolce2*
	regulation of cell proliferation	0.002	*Odc1*, *Nox4*, *Comt*, *Wisp2*, *Sulf1*, *Vsig4*, *Ctgf*, *Sparc*, *Gpx1*, *Serpinf1*, *Nupr1*, *Crlf1*, *Cib1*, *F2r*, *Prnp*, *Sox9*, *Ecm1*
	negative regulation of cell proliferation	0.002	*Nox4*, *Comt*, *Serpinf1*, *Nupr1*, *Cib1*, *Wisp2*, *Sulf1*, *Vsig4*, *F2r*, *Prnp*, *Sox9*
	negative regulation of biological process	0.001	*Plcg2*, *Ptgis*, *Prmt2*, *Lmcd1*, *Kctd1*, *Sulf1*, *Vsig4*, *Phlda3*, *Gpx1*, *Med10*, *Cib1*, *F2r*, *Timp1*, *Sox9*, *Mmp2*, *Ecm1*, *Dpysl3*, *Nox4*, *Anxa5*, *Comt*, *Wisp2*, *Ankrd1*, *Rangap1*, *Cd44*, *Nbl1*, *Serpinf1*, *Nupr1*, *Crlf1*, *Actn4*, *Pde1c*, *Prnp*, *Comp*
	regulation of multicellular organismal process	0.001	*Ptgis*, *Mgp*, *Anxa5*, *Srpx2*, *Anxa3*, *Lmcd1*, *Ankrd1*, *Sulf1*, *Vsig4*, *Cd44*, *Pdlim7*, *Myh7*, *Ctgf*, *Nbl1*, *Gpx1*, *Serpinf1*, *Nupr1*, *F2r*, *Timp1*, *Prnp*, *Sox9*, *Mmp2*, *Ecm1*, *Dpysl3*
	positive regulation of cellular process	0.001	*Ptgis*, *Odc1*, *Prmt2*, *Lmcd1*, *Sulf1*, *Phlda3*, *Gpx1*, *Med10*, *Col8a1*, *F2r*, *Sox9*, *Mmp2*, *Ecm1*, *Dpysl3*, *Fbln2*, *Nox4*, *Anxa5*, *Srpx2*, *Comt*, *Hspa2*, *Anxa3*, *Ankrd1*, *Meox1*, *Cd44*, *Pdlim7*, *Ctgf*, *Nbl1*, *Serpinf1*, *Nupr1*, *Crlf1*, *Actn4*, *Mllt11*
	positive regulation of signal transduction	0.018	*Ptgis*, *Nox4*, *Gpx1*, *Crlf1*, *Lmcd1*, *Ankrd1*, *Sulf1*, *F2r*, *Cd44*, *Ctgf*, *Sox9*, *Ecm1*
	response to stress	0.038	*Ptgis*, *Anxa5*, *Comt*, *Anxa3*, *Lox*, *Hspa2*, *Cd44*, *F13a1*, *Phlda3*, *Gpx1*, *Nupr1*, *Cib1*, *Lyz1*, *Lyz2*, *F2r*, *Ino80c*, *Prnp*, *Mmp2*, *Ecm1*
	extracellular matrix organization	0.002	*Lox*, *Sulf1*, *Mfap5*, *Ctgf*, *Sox9*, *Adamts2*, *Comp*
	sulfur compound metabolic process	0.002	*Chst7*, *Nox4*, *Gpx1*, *Hagh*, *Dcn*, *Sulf1*, *Bgn*
MF	glycosaminoglycan binding	0.001	*Dcn*, *Cd44*, *Pcolce*, *Bgn*, *Prelp*, *Ctgf*, *Comp*, *Pcolce2*, *Dpysl3*, *Fstl1*
	calcium ion binding	0.001	*Mgp*, *Anxa5*, *Anxa3*, *Efhd1*, *S100a11*, *Sulf1*, *Creld1*, *Sparc*, *Actn4*, *Cib1*, *Emr1*, *Cd248*, *Svep1*, *Comp*, *Fstl1*
	extracellular matrix binding	0.002	*Fbln2*, *Dcn*, *Bgn*, *Sparc*, *Ecm1*
	collagen binding	0.023	*Dcn*, *Pcolce*, *Comp*, *Pcolce2*
***Cluster_2***			
BP	cellular modified amino acid metabolic process	0.006	*Gstm1*, *Stat5a*, *Gstk1*, *Mat2a*, *Ccbl2*
	carboxylic acid metabolic process	0.007	*Gstm1*, *Stat5a*, *Gstk1*, *Acot7*, *Mat2a*, *Pkm2*, *Ccbl2*, *Crat*, *Acot1*
	establishment of localization	0.035	*Abcc9*, *Ndufs2*, *Adhfe1*, *Crat*, *Gga2*, *Nudc*, *Fxyd1*, *Ano10*, *Bves*, *Sdhc*, *Tmem38a*, *Agtr1a*, *Stat5a*, *Slc2a4*, *Vdac1*, *Etfb*, *Osbpl3*
MF	transferase activity, transferring alkyl or aryl (other than methyl) groups	0.006	*Gstm1*, *Gstk1*, *Mat2a*, *Gstm7*

GO: Gene Ontology; BP: biological process; MF: molecular function; p_*corr*_: corrected p-value

Quantitative trait analysis showed strong negative correlations between expression levels of several genes in cluster 1 and ipRFAC, with significant correlation coefficients ranging from -0.67 to 0.94 (Table 4). Specifically, an inverse relationship with ipRFAC was observed for: (*a*) ECM genes, included those involved in integrin signaling pathway; (*b*) genes related to cardiac function and repair (cardiovascular system development); (*c*) genes that participate in calcium cell signalling pathways by binding to Ca^2+^; and (*d*) stress response genes, in particular those regulating cell death.

**Table 4:** Correlations between gene expression in specified GO gene sets and ipRFAC.

GO terms	*Gene Symbol*	*r*	p
Integrin-mediated signaling pathway and/or Extracellular matrix organization & binding	*Bgn*	-0.943	< 1 × 10^−7^
	*Itgbl1*	-0.929	1.00 × 10^−7^
	*Ctgf*	-0.922	1.00 × 10^−7^
	*Fbln2*	-0.915	3.00 × 10^−7^
	*Mfap5*	-0.909	5.00 × 10^−7^
	*Adamts2*	-0.888	1.90 × 10^−6^
	*Itga11*	-0.86	9.40 × 10^−6^
	*Ecm1*	-0.812	7.57 × 10^−5^
	*Comp*	-0.812	7.52 × 10^−5^
	*Dcn*	-0.798	1.25 × 10^−4^
	*Sulf1*	-0.759	4.11 × 10^−4^
	*Lox*	-0.756	4.49 × 10^−4^
	*Sparc*	-0.756	4.46 × 10^−4^
	*Cib1*	-0.74	6.86 × 10^−4^
	*Sox9*	-0.676	2.88 × 10^−3^
Cardiovascular system development	*Ctgf*	-0.922	1.00 × 10^−7^
	*Nox4*	-0.886	2.30 × 10^−6^
	*Ankrd1*	-0.884	2.60 × 10^−6^
	*Col8a1*	-0.878	3.60 × 10^−6^
	*Gpx1*	-0.836	2.93 × 10^−5^
	*Srpx2*	-0.826	4.48 × 10^−5^
	*Ecm1*	-0.812	7.57 × 10^−5^
	*Mmp2*	-0.811	7.94 × 10^−5^
	*Myh7*	-0.792	1.48 × 10^−4^
	*Lox*	-0.756	4.49 × 10^−4^
	*Sox9*	-0.676	2.88 × 10^−3^
	*Col4a1*	-0.619	8.05 × 10^−3^
Calcium ion binding	*Svep1*	-0.867	6.80 × 10^−6^
	*Fstl1*	-0.833	3.32 × 10^−5^
	*Anxa3*	-0.823	5.00 × 10^−5^
	*Comp*	-0.812	7.52 × 10^−5^
	*Efhd1*	-0.797	1.27 × 10^−4^
	*Mgp*	-0.793	1.45 × 10^−4^
	*Cd248*	-0.793	1.46 × 10^−4^
	*S100a11*	-0.782	2.08 × 10^−4^
	*Emr1*	-0.773	2.78 × 10^−4^
	*Sulf1*	-0.759	4.11 × 10^−4^
	*Sparc*	-0.756	4.46 × 10^−4^
	*Cib1*	-0.74	6.86 × 10^−4^
	*Creld1*	-0.728	9.22 × 10^−4^
	*Actn4*	-0.722	1.07 × 10^−3^
	*Anxa5*	-0.708	1.48 × 10^−3^
Regulation of cell death	*Ctgf*	-0.922	1.00 × 10^−7^
	*Timp1*	-0.895	1.20 × 10^−6^
	*Nox4*	-0.886	2.30 × 10^−6^
	*Ankrd1*	-0.884	2.60 × 10^−6^
	*Cd44*	-0.849	1.66 × 10^−5^
	*Gpx1*	-0.836	2.93 × 10^−5^
	*Crlf1*	-0.834	3.12 × 10^−5^
	*Plcg2*	-0.832	3.38 × 10^−5^
	*Nupr1*	-0.824	4.84 × 10^−5^
	*Comp*	-0.812	7.52 × 10^−5^
	*Mmp2*	-0.811	7.94 × 10^−5^
	*Phlda3*	-0.8	1.14 × 10^−4^
	*Ptgis*	-0.798	1.23 × 10^−4^
	*Mllt11*	-0.784	1.92 × 10^−4^
	*Prmt2*	-0.777	2.40 × 10^−4^
	*Actn4*	-0.722	1.07 × 10^−3^
	*Prnp*	-0.711	1.38 × 10^−3^
	*Anxa5*	-0.708	1.48 × 10^−3^
	*Sox9*	-0.676	2.88 × 10^−3^
	*F2r*	-0.667	3.45 × 10^−3^
Response to stress	*Comt*	-0.866	7.10 × 10^−6^
	*Cd44*	-0.849	1.66 × 10^−5^
	*Lyz1*	-0.849	1.60 × 10^−5^
	*Gpx1*	-0.836	2.93 × 10^−5^
	*Nupr1*	-0.824	4.84 × 10^−5^
	*Anxa3*	-0.823	5.00 × 10^−5^
	*Lyz2*	-0.814	6.97 × 10^−5^
	*Ecm1*	-0.812	7.57 × 10^−5^
	*Mmp2*	-0.811	7.94 × 10^−5^
	*Phlda3*	-0.8	1.14 × 10^−4^
	*Ptgis*	-0.798	1.23 × 10^−4^
	*Lox*	-0.756	4.49 × 10^−4^
	*Cib1*	-0.74	6.86 × 10^−4^
	*F13a1*	-0.717	1.19 × 10^−3^
	*Prnp*	-0.711	1.38 × 10^−3^
	*Anxa5*	-0.708	1.48 × 10^−3^
	*Hspa2*	-0.705	1.56 × 10^−3^
	*Ino80c*	-0.685	2.39 × 10^−3^
	*F2r*	-0.667	3.45 × 10^−3^

*r*: correlation coefficient; p: parametric p-value

## Discussion

Cardiac magnetic resonance imaging in cardiovascular research is generally used to measure myocardial mass, LV volumes, or EF. Although such measures of global function are very useful, in the MI setting, where contractile dysfunction is generally localized to specific coronary territories, it is critically important to evaluate regional myocardial function.

We already reported the CMR regional quantification of LV function by RFAC analysis where the LV cavity was divided into several regions and the RFAC computed for each sector and clearly visualized in a "bull’s eye" format [12]. This approach, allowing the non-invasive localization of the infarction and the accurate functional characterization of the post-ischemic cardiac remodeling, has potential application for assessing the effectiveness of pharmacological treatments or regenerative medicine interventions. To explore this idea, we investigated the ability of RFAC in discriminating the effects of treatment with valsartan, a selective inhibitor of AT1R, on LV regional endocardial wall motion and systolic function in mouse model of MI. The choice of valsartan was motivated by the role of the renin—angiotensin—aldosterone system (RAAS) in cardiovascular disease, whose activation results in increased serum level of angiotensin II that perpetuates vasoconstriction, endothelial dysfunction, LV hypertrophy, myocardial fibrosis and remodeling [24–26]. Modulation of the RAAS has long been acknowledged as a target of pharmacological treatment for primary and secondary cardiovascular prevention. Clinical evidence showed that long-term administration of RAAS blockers, such as ACE inhibitors and angiotensin receptor blockers, plays a critical role in post-infarction LV remodeling attenuating fibrosis in both infarcted and non-infarcted myocardium and consequently promoting the improvement of heart function and survival [27, 28].

This paper, while confirming that RFAC is a sensitive and accurate tool for LV endocardial wall motion characterization [12], clearly highlights the effectiveness of this regional index, compared to global parameters, in discriminating qualitatively and quantitatively the effects of pharmacological intervention on cardiac performance.

Throughout the follow-up, bull’s eye visualization showed that anterior myocardial infarction resulted in acute loss of LV systolic function and progression of LV impairment. The expansion of damaged myocardium affected not only apical and anterior sectors, but also the inferior and posterior mid-papillary regions, indicating a progressive loss of contraction in remote non-infarcted tissue probably as a result of LV stiffness and remodeling. Most interestingly, RFAC analysis accurately located the regions where valsartan exerted its protective effect, highlighting the drug efficacy in improving contractile function and reducing maladaptive remodeling. At 1-week post surgery, when remodeling was still progressing, even in the presence of many processes that could act as confounding factors (such as inflammation, angiogenesis and granulation tissue formation), RFAC was able to show significant differences in mid-apical regions between MI and MI+Val groups. At 4 weeks, when the remodeling was almost complete, differences between groups became even more significant and localized mostly in the mid apical free wall region. Tissue analysis of these latter zones showed, at the cellular level, attenuated myocyte hypertrophy, reduction in number of proliferating fibroblast and decreased deposition of interstitial collagen, which were associated with an overall beneficial effect on ventricular remodeling and with improved heart function.

The protective effect documented by CMR is consistent with results from genome-wide gene expression profiling. This analysis shows that valsartan exerted its function by modulating target genes involved in several processes such as cell death and proliferation, Ca^2+^ binding, integrin-mediated signaling, ECM organization and response to stress, which were significantly altered by infarct and almost restored to the normal level by valsartan treatment (Fig 6). Coherently, several genes, belonging to the above biological processes and molecular functions, showed an inverse correlation with RFAC, *i*.*e*. the greater the LV regional wall motion and contraction, the lower their expression. Indeed, cardiac development, Ca^2+^ signaling, ECM, and stress response related genes showed higher expression in the LV remote non-infarcted free wall of sham mice, intermediate in the valsartan-treated MI group, and lower in untreated MI mice, suggesting that infaction induces compensatory/repair pathways which are attenuated by valsartan protective effects.

Importantly, genes playing key roles in the development of the pro-fibrotic process were massively upregulated in remote non-infarcted myocardium in the MI group and, consistent with a recent report [29], significantly modulated by Valsartan. In particular, post-MI remodeling was associated with a complex response of genes encoding ECM-related molecules, such as Tissue Inhibitor of Metalloproteinases-1 (TIMP1) and Fibulin 2. Elevated TIMPs have been demonstrated in the infarcted heart [30, 31] and contributed to collagen accumulation in the process of scar formation probably by stimulating fibroblast growth [30, 32, 33]. Thus, TIMP1 over-expression may favor cardiac fibrosis not only by inhibiting matrix metalloproteinase, but also by independent direct pro-fibrotic mechanisms. Fibulin 2 is an ECM protein that can interact with a wide range of ECM proteins and be incorporated into various extracellular structures. A number of observations [34, 35] suggest that Fibulin 2 plays significant roles in embryonic development and tissue remodeling. Following valsartan treatment, this group of genes was negatively regulated. This data, together with the evidence of the anti-hypertrophic and anti-fibrotic effects observed by tissue analysis, led us to hypothesize that these factors likely participate in convergent pathways. Further investigation will delineate their specific contributions and may provide novel therapeutic targets.

Clinical evidences show that infarction affects both LV and LA and the ability of the latter to respond to a decrease in LV compliance by increasing its size [36]. Indeed, after infarction, LV end-diastolic pressure and wall tension increase [37] and, consequently, LA enlarges to maintain adequate LV filling: these modifications, indexed by LA volume quantitation, can be used as a sensitive and specific tool for the detection of LV dysfunction [16, 38, 39]. The beneficial effects on LV global volumes and RFAC exerted by valsartan were confirmed by the echocardiographic analysis of LA volume. Concurrently to LA enlargements, also LA appendage increased in lengths and this process appeared to progress over time. Tissue analyses revealed that the increase in dimension was accompanied to an increase in fibrosis in MI group, probably as direct consequence of LV dysfunction and hemodynamic atrial overload [40, 41]. Treatment with valsartan attenuated LV dysfunction, and this was associated with regression of atrial and appendage myocardium remodeling.

LA volume increase reflects the average effect of LV filling pressures over time [36, 42] and is an index of ventricular dysfunction. We also observe that, for murine MI model, the LA volume increase is a more sensitive marker of LV dysfunction than the posterior wall thickness, which showed only minimal changes.

In summary, our study provides strong evidence that in the evaluation of a pharmacological therapy following infarction, RFAC can detect (and localize) much subtler changes in cardiac performance than EF, which is the weighted average of the RFAC on the whole ventricle volume. Comparing RFAC with strain echocardiography could add more information on regional function and/or the benefits of RFAC, but unfortunately this echocardiographic tool is unavailable to us.

## Conclusions

RFAC index accurately monitored the efficacy of a pharmacological treatment during the follow-up and indicated the inferior and posterior sectors of the LV remote non-infarcted free wall as those where valsartan exerted a protective effect on contraction. This data was supported by evidences at the cellular level indicating attenuated myocyte hypertrophy, reduction in number of proliferating fibroblast and decreased deposition of fibrillar collagen, and by modulation of relevant gene pathways. The overall beneficial effect on ventricular remodeling was additionally confirmed by attenuation of LA enlargement.

Taken together, these data indicate that RFAC index could be applied in studies assessing localized and global wall motion differences secondary to standard pharmacological treatment in a mouse model of infarction.

This information might serve to provide a novel view on the evaluation of tissue injury and its response to ischemia, and contribute to gain new insights into the evaluation of new therapies, which in the future could be translated to acute MI patients.

## Supporting Information

S1 Table LV structure and function by Echocardiography. (DOCX)

S2 Table List of differentially expressed genes. (DOCX)
