# Supplementary material for: Evaluation of Left Ventricle Function by Regional Fractional Area Change (RFAC) in a Mouse Model of Myocardial Infarction Secondary to Valsartan Treatment
Source: PLoS One. 2015 Aug 20;10(8):e0135778. doi: 10.1371/journal.pone.0135778 (PMC4546366; doi:10.1371/journal.pone.0135778)
Supplement: S1 Table — (DOCX) [file pone.0135778.s001.docx]

**S1 Table.** **LV structure and function by Echo.**

|  | Mice | LV EDV  (μl) | LV ESV  (μl) | LV SV  (μl) | LV EF  (%) | LV mass  (mg) | PWth (D)  (mm) |  |
| --- | --- | --- | --- | --- | --- | --- | --- | --- |
| **Baseline** | 12 | 40± 2 | 9 ± 2 | 30 ± 1 | 76 ± 3 | 87 ± 4 | 0.78 ± 0.02 |  |
| **24h** | | | | | | | | |
| Sham | 12 | 41 ± 2 | 12 ± 1 | 29 ± 2 | 70 ± 3 | 85 ± 4 | 0.78 ± 0.03 |  |
| MI and MI+Val | 24 | 64 ± 3^§§§^ | 39 ± 2^§§§^ | 25 ± 2 | 38 ± 1^§§§^ | 103 ± 7 | 0.79 ± 0.03 |  |
| MI | 12 | 66 ± 4 | 40 ± 3 | 26 ± 2 | 40 ± 2 | 106 ± 10 | 0.79 ± 0.05 |  |
| MI+Val | 12 | 62 ± 5 | 39 ± 3 | 23 ± 2 | 37 ± 2 | 101 ± 9 | 0.78 ± 0.04 |  |
| **1w** | | | | | | | | |
| Sham | 12 | 43 ± 2 | 10 ± 1 | 32 ± 2 | 76 ± 3 | 85 ± 3 | 0.84 ± 0.03 |  |
| MI | 12 | 101 ± 11^§§§^ | 67 ± 9^§§§^ | 34 ± 4 | 34 ± 4^§§§^ | 125 ± 14^§§§^ | 0.97 ± 0.05^§^ |  |
| MI+Val | 12 | 76 ± 4^*^ | 45± 4^**^ | 31 ± 2 | 41 ± 3 | 128 ± 11 | 0.81 ± 0.05^*^ |  |
| **4w** | | | | | | | | |
| Sham | 12 | 45 ± 2 | 12 ± 2 | 34 ± 1 | 75 ± 3 | 94± 4 | 0.91 ± 0.05 |  |
| MI | 12 | 130 ± 11^§§§^ | 92 ± 9^§§§^ | 38 ± 3 | 30 ± 2^§§§^ | 175 ± 14^§§§^ | 1.06 ± 0.05^§^ |  |
| MI+Val | 12 | 97 ± 5^**^ | 63 ± 4^**^ | 33 ± 3 | 35 ± 3 | 134 ± 8^**^ | 0.82 ± 0.03^**^ |  |

Echocardiographic evaluation of LV structure and function at the baseline and in sham, MI and MI+Val groups during the follow-up period. Values are presented as mean ± SEM. ^*^ p<0.05 and ^**^ p<0.01 *vs* MI. § p<0.05 and §§§ p<0.001 *vs* Sham.
