# Supplementary material for: Evaluation of Left Ventricle Function by Regional Fractional Area Change (RFAC) in a Mouse Model of Myocardial Infarction Secondary to Valsartan Treatment
Source: PLoS One. 2015 Aug 20;10(8):e0135778. doi: 10.1371/journal.pone.0135778 (PMC4546366; doi:10.1371/journal.pone.0135778)
Supplement: S2 Table — (DOCX) [file pone.0135778.s002.docx]

**S2 Table.** Whole-genome gene expression analysis.

|  |  |  |  | **Fold Change** | | **Pairwise comparison** | |
| --- | --- | --- | --- | --- | --- | --- | --- |
| ***Gene Symbol*** | **Gene Name** | **EntrezID** | **P-value** | **MI/Sham FC** | **MI+VAL/MI FC** | **MI/Sham** | **MI+VAL/MI** |
| *Abcc9* | ATP-binding cassette, sub-family C (CFTR/MRP), member 9 | 20928 | 0.0011554 | -1.30 | 1.21 | ** | ** |
| *Acot1* | acyl-CoA thioesterase 1 | 26897 | 0.0002370 | -2.19 | 1.18 | ** |  |
| *Acot7* | acyl-CoA thioesterase 7 | 70025 | 0.0004932 | -1.29 | 1.10 | ** |  |
| *Acta1* | actin, alpha 1, skeletal muscle | 11459 | 0.0000492 | 3.96 | -1.81 | ** |  |
| *Actn4* | actinin alpha 4 | 60595 | 0.0005433 | 1.25 | -1.05 | ** |  |
| *Adamts2* | a disintegrin-like and metallopeptidase (reprolysin type) with thrombospondin type 1 motif, 2 | 216725 | 0.0000036 | 1.79 | -1.33 | ** | ** |
| *Adhfe1* | alcohol dehydrogenase, iron containing, 1 | 76187 | 0.0008336 | -1.25 | 1.06 | ** |  |
| *Aes* | amino-terminal enhancer of split | 14797 | 0.0001722 | -1.20 | 1.11 | ** | ** |
| *Agtr1a* | angiotensin II receptor, type 1a | 11607 | 0.0008910 | -1.22 | 1.13 | ** | ** |
| *Amy2a5* | amylase 2a5 | 109959 | 0.0002348 | -1.30 | 1.07 | ** |  |
| *Ankrd1* | ankyrin repeat domain 1 (cardiac muscle) | 107765 | 0.0000990 | 2.88 | -1.41 | ** |  |
| *Ano10* | anoctamin 10 | 102566 | 0.0000019 | -2.42 | 1.38 | ** | ** |
| *Anxa3* | annexin A3 | 11745 | 0.0000582 | 1.49 | -1.25 | ** | ** |
| *Anxa5* | annexin A5 | 11747 | 0.0015636 | 1.31 | -1.21 | ** | ** |
| *Apbb1* | amyloid beta (A4) precursor protein-binding, family B, member 1 | 11785 | 0.0000008 | -1.58 | 1.23 | ** | ** |
| *Aph1a* | anterior pharynx defective 1a homolog (C. elegans) | 226548 | 0.0010456 | -1.32 | 1.06 | ** |  |
| *Arpc5* | actin related protein 2/3 complex, subunit 5 | 67771 | 0.0003310 | 1.32 | -1.26 | ** | ** |
| *Bgn* | biglycan | 12111 | 0.0000022 | 1.95 | -1.30 | ** | ** |
| *Bves* | blood vessel epicardial substance | 23828 | 0.0000267 | -1.34 | 1.15 | ** | ** |
| *Ccbl2* | cysteine conjugate-beta lyase 2 | 229905 | 0.0012958 | -1.22 | 1.07 | ** |  |
| *Cd248* | CD248 antigen, endosialin | 70445 | 0.0000586 | 1.41 | -1.22 | ** | ** |
| *Cd44* | CD44 antigen | 12505 | 0.0000826 | 1.39 | -1.16 | ** |  |
| *Ces1d* | carboxylesterase 1D | 104158 | 0.0002380 | -2.43 | 1.62 | ** |  |
| *Chmp4b* | chromatin modifying protein 4B | 75608 | 0.0008323 | 1.28 | -1.10 | ** |  |
| *Chst7* | carbohydrate (N-acetylglucosamino) sulfotransferase 7 | 60322 | 0.0002045 | 1.21 | -1.15 | ** | ** |
| *Cib1* | calcium and integrin binding 1 (calmyrin) | 23991 | 0.0013235 | 1.34 | -1.17 | ** |  |
| *Ckb* | creatine kinase, brain | 12709 | 0.0003556 | 1.44 | -1.12 | ** |  |
| *Cnst* | consortin, connexin sorting protein | 226744 | 0.0000243 | -1.34 | 1.19 | ** | ** |
| *Col16a1* | collagen, type XVI, alpha 1 | 107581 | 0.0002500 | 1.42 | -1.25 | ** | ** |
| *Col4a1* | collagen, type IV, alpha 1 | 12826 | 0.0013718 | 1.36 | 1.02 | ** |  |
| *Col8a1* | collagen, type VIII, alpha 1 | 12837 | 0.0000003 | 2.07 | -1.54 | ** | ** |
| *Comp* | cartilage oligomeric matrix protein | 12845 | 0.0002911 | 1.96 | -1.52 | ** | ** |
| *Comt* | catechol-O-methyltransferase | 12846 | 0.0000247 | 1.35 | -1.04 | ** |  |
| *Coq10a* | coenzyme Q10 homolog A (yeast) | 210582 | 0.0002407 | -1.25 | 1.16 | ** | ** |
| *Crat* | carnitine acetyltransferase | 12908 | 0.0001655 | -1.27 | 1.10 | ** |  |
| *Creld1* | cysteine-rich with EGF-like domains 1 | 171508 | 0.0016824 | 1.23 | -1.14 | ** |  |
| *Crlf1* | cytokine receptor-like factor 1 | 12931 | 0.0000357 | 1.25 | -1.16 | ** | ** |
| *Ctgf* | connective tissue growth factor | 14219 | 0.0001623 | 2.72 | -1.55 | ** |  |
| *Cutc* | cutC copper transporter homolog (E.coli) | 66388 | 0.0000073 | -1.23 | 1.02 | ** |  |
| *Cyp27a1* | cytochrome P450, family 27, subfamily a, polypeptide 1 | 104086 | 0.0005890 | 1.27 | -1.07 | ** |  |
| *Dcn* | decorin | 13179 | 0.0004675 | 1.37 | -1.09 | ** |  |
| *Dhrs11* | dehydrogenase/reductase (SDR family) member 11 | 192970 | 0.0007368 | -1.26 | 1.13 | ** |  |
| *Dpysl3* | dihydropyrimidinase-like 3 | 22240 | 0.0019402 | 1.21 | -1.21 | ** | ** |
| *Ecm1* | extracellular matrix protein 1 | 13601 | 0.0005592 | 1.32 | -1.10 | ** |  |
| *Efhd1* | EF hand domain containing 1 | 98363 | 0.0005240 | 1.37 | -1.13 | ** |  |
| *Emp1* | epithelial membrane protein 1 | 13730 | 0.0000033 | 1.82 | -1.54 | ** | ** |
| *Emr1* | EGF-like module containing, mucin-like, hormone receptor-like sequence 1 | 13733 | 0.0014563 | 1.30 | -1.06 | ** |  |
| *Endod1* | endonuclease domain containing 1 | 71946 | 0.0000097 | 1.33 | -1.26 | ** | ** |
| *Etfb* | electron transferring flavoprotein, beta polypeptide | 110826 | 0.0004748 | -1.22 | 1.02 | ** |  |
| *F13a1* | coagulation factor XIII, A1 subunit | 74145 | 0.0006690 | 1.38 | -1.21 | ** |  |
| *F2r* | coagulation factor II (thrombin) receptor | 14062 | 0.0005839 | 1.24 | -1.22 | ** | ** |
| *Fbln2* | fibulin 2 | 14115 | < 0.0000001 | 1.94 | -1.34 | ** | ** |
| *Flcn* | folliculin | 216805 | 0.0012066 | -1.34 | 1.10 | ** |  |
| *Fstl1* | follistatin-like 1 | 14314 | 0.0000909 | 1.68 | -1.33 | ** | ** |
| *Fxyd1* | FXYD domain-containing ion transport regulator 1 | 56188 | 0.0000705 | -1.28 | 1.13 | ** | ** |
| *Fxyd6* | FXYD domain-containing ion transport regulator 6 | 59095 | 0.0000141 | 1.84 | -1.28 | ** |  |
| *Gcat* | glycine C-acetyltransferase (2-amino-3-ketobutyrate-coenzyme A ligase) | 26912 | 0.0001174 | -1.33 | 1.15 | ** |  |
| *Gga2* | golgi associated, gamma adaptin ear containing, ARF binding protein 2 | 74105 | 0.0006290 | -1.28 | 1.06 | ** |  |
| *Gm5512* | required for meiotic nuclear division 1 pseudogene | 433224 | 0.0009128 | -1.27 | 1.21 | ** | ** |
| *Gpt* | glutamic pyruvic transaminase, soluble | 76282 | 0.0000616 | -1.38 | 1.23 | ** | ** |
| *Gpx1* | glutathione peroxidase 1 | 14775 | 0.0001937 | 1.55 | -1.25 | ** |  |
| *Gstk1* | glutathione S-transferase kappa 1 | 76263 | 0.0012501 | -1.34 | 1.22 | ** |  |
| *Gstm1* | glutathione S-transferase, mu 1 | 14862 | 0.0000272 | -1.38 | 1.13 | ** |  |
| *Gstm7* | glutathione S-transferase, mu 7 | 68312 | 0.0010126 | -1.28 | 1.14 | ** |  |
| *Hagh* | hydroxyacyl glutathione hydrolase | 14651 | 0.0000074 | 1.30 | -1.08 | ** |  |
| *Hn1* | hematological and neurological expressed sequence 1 | 15374 | 0.0005499 | 1.54 | -1.19 | ** |  |
| *Hrc* | histidine rich calcium binding protein | 15464 | 0.0000811 | -1.37 | 1.17 | ** | ** |
| *Hs3st1* | heparan sulfate (glucosamine) 3-O-sulfotransferase 1 | 15476 | 0.0015848 | 1.22 | -1.25 | ** | ** |
| *Hspa2* | heat shock protein 2 | 15512 | 0.0016023 | 1.32 | -1.09 | ** |  |
| *Ift81* | intraflagellar transport 81 homolog (Chlamydomonas) | 12589 | 0.0002838 | -1.56 | 1.39 | ** | ** |
| *Igfbp7* | insulin-like growth factor binding protein 7 | 29817 | 0.0009493 | 1.44 | -1.11 | ** |  |
| *Igsf1* | immunoglobulin superfamily, member 1 | 209268 | 0.0000048 | 1.61 | -1.23 | ** | ** |
| *Ino80c* | INO80 complex subunit C | 225280 | 0.0001275 | 1.20 | -1.15 | ** | ** |
| *Itga11* | integrin alpha 11 | 319480 | 0.0008922 | 1.23 | -1.07 | ** |  |
| *Itga7* | integrin alpha 7 | 16404 | 0.0000812 | -1.31 | 1.09 | ** |  |
| *Itgbl1* | integrin, beta-like 1 | 223272 | 0.0000007 | 1.79 | -1.44 | ** | ** |
| *Kctd1* | potassium channel tetramerisation domain containing 1 | 106931 | 0.0019543 | 1.28 | -1.10 | ** |  |
| *Khdrbs3* | KH domain containing, RNA binding, signal transduction associated 3 | 13992 | 0.0005721 | -1.20 | 1.14 | ** | ** |
| *Lgr6* | leucine-rich repeat-containing G protein-coupled receptor 6 | 329252 | 0.0002972 | -1.23 | 1.07 | ** |  |
| *Lman1l* | lectin, mannose-binding 1 like | 235416 | 0.0018026 | 1.25 | -1.19 | ** | ** |
| *Lmcd1* | LIM and cysteine-rich domains 1 | 30937 | 0.0011656 | 1.36 | -1.13 | ** |  |
| *LOC100047583* | apolipoprotein D-like | 100047583 | 0.0007742 | 1.51 | -1.35 | ** | ** |
| *Lox* | lysyl oxidase | 16948 | 0.0010176 | 1.21 | -1.15 | ** | ** |
| *Loxl1* | lysyl oxidase-like 1 | 16949 | 0.0000114 | 1.86 | -1.25 | ** |  |
| *Lrg1* | leucine-rich alpha-2-glycoprotein 1 | 76905 | 0.0000681 | -1.24 | 1.03 | ** |  |
| *Lyz1* | lysozyme 1 | 17110 | 0.0001749 | 1.55 | -1.25 | ** |  |
| *Lyz2* | lysozyme 2 | 17105 | 0.0006210 | 1.69 | -1.45 | ** | ** |
| *Mat2a* | methionine adenosyltransferase II, alpha | 232087 | 0.0002439 | -1.29 | 1.24 | ** | ** |
| *Med10* | mediator of RNA polymerase II transcription, subunit 10 homolog (NUT2, S. cerevisiae) | 28077 | 0.0000672 | 1.31 | -1.09 | ** |  |
| *Meox1* | mesenchyme homeobox 1 | 17285 | 0.0000717 | 1.85 | -1.33 | ** |  |
| *Mfap4* | microfibrillar-associated protein 4 | 76293 | 0.0000437 | 1.96 | -1.23 | ** |  |
| *Mfap5* | microfibrillar associated protein 5 | 50530 | 0.0000025 | 2.75 | -1.82 | ** | ** |
| *Mgp* | matrix Gla protein | 17313 | 0.0015754 | 1.56 | -1.34 | ** |  |
| *Mllt11* | myeloid/lymphoid or mixed-lineage leukemia (trithorax homolog, Drosophila); translocated to, 11 | 56772 | 0.0002441 | 1.34 | -1.21 | ** | ** |
| *Mmp2* | matrix metallopeptidase 2 | 17390 | 0.0000984 | 1.37 | -1.14 | ** |  |
| *Mmp23* | matrix metallopeptidase 23 | 26561 | 0.0015590 | 1.27 | -1.11 | ** |  |
| *Mrm1* | mitochondrial rRNA methyltransferase 1 homolog (S. cerevisiae) | 217038 | 0.0015495 | -1.38 | 1.11 | ** |  |
| *Mrpl37* | mitochondrial ribosomal protein L37 | 56280 | 0.0015403 | -1.21 | 1.12 | ** |  |
| *Mxra8* | matrix-remodelling associated 8 | 74761 | 0.0002413 | 1.31 | -1.19 | ** | ** |
| *Myh7* | myosin, heavy polypeptide 7, cardiac muscle, beta | 140781 | 0.0017167 | 1.75 | -1.47 | ** |  |
| *Myh8* | myosin, heavy polypeptide 8, skeletal muscle, perinatal | 17885 | 0.0010867 | -1.35 | 1.26 | ** | ** |
| *Nav1* | neuron navigator 1 | 215690 | 0.0000775 | 1.33 | -1.17 | ** | ** |
| *Nbl1* | neuroblastoma, suppression of tumorigenicity 1 | 17965 | 0.0014327 | 1.41 | -1.21 | ** |  |
| *Ndufs2* | NADH dehydrogenase (ubiquinone) Fe-S protein 2 | 226646 | 0.0016591 | -1.22 | 1.09 | ** |  |
| *Nhsl1* | NHS-like 1 | 215819 | 0.0000316 | -1.26 | 1.14 | ** | ** |
| *Nox4* | NADPH oxidase 4 | 50490 | 0.0000343 | 1.24 | -1.15 | ** | ** |
| *Nudc* | nuclear distribution gene C homolog (Aspergillus) | 18221 | 0.0000478 | -1.58 | 1.25 | ** | ** |
| *Nupr1* | nuclear protein 1 | 56312 | 0.0007776 | 1.60 | -1.35 | ** |  |
| *Odc1* | ornithine decarboxylase, structural 1 | 18263 | 0.0000857 | 1.23 | -1.25 | ** | ** |
| *Ogfrl1* | opioid growth factor receptor-like 1 | 70155 | 0.0000424 | 1.29 | -1.13 | ** | ** |
| *Osbpl3* | oxysterol binding protein-like 3 | 71720 | 0.0006348 | -1.23 | 1.14 | ** | ** |
| *Palmd* | palmdelphin | 114301 | 0.0005026 | 1.26 | -1.06 | ** |  |
| *Pamr1* | peptidase domain containing associated with muscle regeneration 1 | 210622 | 0.0000985 | 1.41 | -1.26 | ** | ** |
| *Pcolce* | procollagen C-endopeptidase enhancer protein | 18542 | 0.0006214 | 1.45 | -1.22 | ** |  |
| *Pcolce2* | procollagen C-endopeptidase enhancer 2 | 76477 | 0.0011741 | 1.42 | -1.12 | ** |  |
| *Pde1c* | phosphodiesterase 1C | 18575 | 0.0006137 | 1.28 | -1.11 | ** |  |
| *Pdk2* | pyruvate dehydrogenase kinase, isoenzyme 2 | 18604 | 0.0013060 | -1.41 | 1.23 | ** |  |
| *Pdlim7* | PDZ and LIM domain 7 | 67399 | 0.0017147 | 1.24 | -1.05 | ** |  |
| *Phkg1* | phosphorylase kinase gamma 1 | 18682 | 0.0015525 | -1.26 | 1.28 | ** | ** |
| *Phlda3* | pleckstrin homology-like domain, family A, member 3 | 27280 | 0.0009551 | 1.27 | -1.11 | ** |  |
| *Pi16* | peptidase inhibitor 16 | 74116 | 0.0000913 | 1.82 | -1.45 | ** | ** |
| *Pkm2* | pyruvate kinase, muscle | 18746 | 0.0008599 | -1.20 | 1.15 | ** | ** |
| *Pla1a* | phospholipase A1 member A | 85031 | 0.0010786 | 1.28 | -1.17 | ** |  |
| *Plcg2* | phospholipase C, gamma 2 | 234779 | 0.0002872 | 1.29 | -1.15 | ** |  |
| *Pon2* | paraoxonase 2 | 330260 | 0.0012042 | 1.22 | -1.14 | ** |  |
| *Popdc2* | popeye domain containing 2 | 64082 | 0.0007509 | 1.20 | -1.13 | ** | ** |
| *Ppap2c* | phosphatidic acid phosphatase type 2C | 50784 | 0.0001334 | 1.37 | -1.12 | ** |  |
| *Ppic* | peptidylprolyl isomerase C | 19038 | 0.0012905 | 1.38 | -1.18 | ** |  |
| *Pqlc3* | PQ loop repeat containing | 217430 | 0.0000029 | 1.39 | -1.26 | ** | ** |
| *Prelp* | proline arginine-rich end leucine-rich repeat | 116847 | 0.0003133 | 1.57 | -1.23 | ** |  |
| *Prmt2* | protein arginine N-methyltransferase 2 | 15468 | 0.0009167 | 1.24 | -1.12 | ** |  |
| *Prnp* | prion protein | 19122 | 0.0006640 | 1.31 | -1.24 | ** | ** |
| *Ptgis* | prostaglandin I2 (prostacyclin) synthase | 19223 | 0.0003432 | 1.60 | -1.39 | ** | ** |
| *Pxmp2* | peroxisomal membrane protein 2 | 19301 | 0.0002866 | -1.28 | 1.20 | ** | ** |
| *Rangap1* | RAN GTPase activating protein 1 | 19387 | 0.0000561 | 1.27 | -1.16 | ** | ** |
| *Rdm1* | RAD52 motif 1 | 66599 | 0.0017893 | -1.26 | 1.10 | ** |  |
| *Rnase4* | ribonuclease, RNase A family 4 | 58809 | 0.0019567 | 1.28 | -1.29 | ** | ** |
| *Rps6* | ribosomal protein S6 | 20104 | 0.0000238 | -1.27 | -1.02 | ** |  |
| *Rrp12* | ribosomal RNA processing 12 homolog (S. cerevisiae) | 107094 | 0.0003542 | 1.47 | -1.18 | ** |  |
| *S100a11* | S100 calcium binding protein A11 (calgizzarin) | 20195 | 0.0000880 | 1.34 | -1.19 | ** | ** |
| *Scd4* | stearoyl-coenzyme A desaturase 4 | 329065 | 0.0011193 | 2.19 | -1.47 | ** |  |
| *Sdhc* | succinate dehydrogenase complex, subunit C, integral membrane protein | 66052 | 0.0009306 | -1.20 | 1.05 | ** |  |
| *Serpinf1* | serine (or cysteine) peptidase inhibitor, clade F, member 1 | 20317 | 0.0000055 | 2.10 | -1.57 | ** | ** |
| *Slc2a4* | solute carrier family 2 (facilitated glucose transporter), member 4 | 20528 | 0.0009161 | -1.40 | 1.13 | ** |  |
| *Sox9* | SRY-box containing gene 9 | 20682 | 0.0002009 | 1.36 | -1.22 | ** | ** |
| *Sparc* | secreted acidic cysteine rich glycoprotein | 20692 | 0.0002420 | 1.75 | -1.40 | ** | ** |
| *Srpx2* | sushi-repeat-containing protein, X-linked 2 | 68792 | 0.0001483 | 1.38 | -1.20 | ** | ** |
| *Stat5a* | signal transducer and activator of transcription 5A | 20850 | 0.0002509 | -1.22 | 1.11 | ** |  |
| *Sulf1* | sulfatase 1 | 240725 | 0.0000018 | 1.28 | -1.27 | ** | ** |
| *Svep1* | sushi, von Willebrand factor type A, EGF and pentraxin domain containing 1 | 64817 | 0.0001711 | 1.43 | -1.18 | ** |  |
| *Timp1* | tissue inhibitor of metalloproteinase 1 | 21857 | 0.0000197 | 2.41 | -1.63 | ** | ** |
| *Tmem38a* | transmembrane protein 38A | 74166 | 0.0008636 | -1.33 | 1.17 | ** |  |
| *Tspan17* | tetraspanin 17 | 74257 | 0.0000270 | 1.55 | -1.18 | ** |  |
| *Uck2* | uridine-cytidine kinase 2 | 80914 | 0.0001513 | 1.72 | -1.49 | ** | ** |
| *Vdac1* | voltage-dependent anion channel 1 | 22333 | 0.0004703 | -1.36 | 1.24 | ** | ** |
| *Vsig4* | V-set and immunoglobulin domain containing 4 | 278180 | 0.0002821 | 1.21 | -1.19 | ** | ** |
| *Whrn* | whirlin | 73750 | 0.0000459 | -1.56 | 1.34 | ** | ** |
| *Wisp2* | WNT1 inducible signaling pathway protein 2 | 22403 | 0.0000093 | 3.15 | -1.88 | ** | ** |
| *Xirp2* | xin actin-binding repeat containing 2 | 241431 | 0.0003917 | 1.40 | -1.15 | ** |  |
| *Zfp787* | zinc finger protein 787 | 67109 | 0.0008359 | -1.24 | 1.13 | ** |  |
| *1110003E01Rik* | RIKEN cDNA 1110003E01 gene | 68552 | 0.0004024 | 1.29 | -1.16 | ** |  |
| *1190003J15Rik* | RIKEN cDNA 1190003J15 gene | 76974 | 0.0009669 | 1.21 | -1.07 | ** |  |
| *1600021P15Rik* | RIKEN cDNA 1600021P15 gene | 239796 | 0.0018821 | -1.26 | 1.16 | ** |  |
| *1700025G04Rik* | RIKEN cDNA 1700025G04 gene | 69399 | 0.0005679 | 1.36 | -1.14 | ** |  |
| *1810011O10Rik* | RIKEN cDNA 1810011O10 gene | 69068 | 0.0002731 | -1.22 | 1.30 | ** | ** |
| *1810055G02Rik* | RIKEN cDNA 1810055G02 gene | 72056 | 0.0001921 | 1.28 | -1.21 | ** | ** |
| *2310067B10Rik* | RIKEN cDNA 2310067B10 gene | 71947 | 0.0017939 | -1.20 | 1.14 | ** |  |
| *2610039C10Rik* | RIKEN cDNA 2610039C10 gene | 66578 | 0.0003746 | 1.21 | -1.19 | ** | ** |
| *2610528E23Rik* | RIKEN cDNA 2610528E23 gene | 66497 | 0.0000516 | -1.68 | 1.31 | ** | ** |
| *3110040M04Rik* | RIKEN cDNA 3110040M04 gene | 73176 | 0.0009817 | 1.47 | -1.22 | ** |  |
| *3632451O06Rik* | RIKEN cDNA 3632451O06 gene | 67419 | 0.0016412 | -1.35 | 1.11 | ** |  |
| *4930534B04Rik* | RIKEN cDNA 4930534B04 gene | 75216 | 0.0000634 | -1.31 | 1.14 | ** | ** |
| *6330406I15Rik* | RIKEN cDNA 6330406I15 gene | 70717 | 0.0013106 | 1.38 | -1.30 | ** | ** |
| *A830073O21Rik* | RIKEN cDNA A830073O21 gene | 320262 | 0.0005587 | -1.22 | 1.05 | ** |  |
| Whole-genome gene expression analysis in sham, MI and MI+Val groups. List shows genes significatively modulated by myocardial infarction with a significant difference (*p*<0.01, FC ≥ ±1.2) compare to sham and modulation exerted by valsartan treatment. Post-hoc pairwise comparisons (MI *vs.* Sham and MI+Val *vs.* MI). | | | | | | | |
